# A spatiotemporal reconstruction of daily ambient temperature using satellite data in the Megalopolis of Central Mexico from 2003 to 2019

**DOI:** 10.1002/joc.7060

**Published:** 2021-03-18

**Authors:** Iván Gutiérrez‐Avila, Kodi B. Arfer, Sandy Wong, Johnathan Rush, Itai Kloog, Allan C. Just

**Affiliations:** ^1^ Department of Environmental Medicine and Public Health Icahn School of Medicine at Mount Sinai New York New York USA; ^2^ Department of Geography Florida State University (FSU) Tallahassee Florida USA; ^3^ Department of Geography and Environmental Development Ben‐Gurion University of the Negev Beersheba Israel

**Keywords:** extreme air temperature, human exposure, land surface temperature, Megalopolis of Central Mexico, MODIS, remote sensing

## Abstract

While weather stations generally capture near‐surface ambient air temperature (Ta) at a high temporal resolution to calculate daily values (i.e., daily minimum, mean, and maximum Ta), their fixed locations can limit their spatial coverage and resolution even in densely populated urban areas. As a result, data from weather stations alone may be inadequate for Ta‐related epidemiology particularly when the stations are not located in the areas of interest for human exposure assessment. To address this limitation in the Megalopolis of Central Mexico (MCM), we developed the first spatiotemporally resolved hybrid satellite‐based land use regression Ta model for the region, home to nearly 30 million people and includes Mexico City and seven more metropolitan areas. Our model predicted daily minimum, mean, and maximum Ta for the years 2003–2019. We used data from 120 weather stations and Land Surface Temperature (LST) data from NASA's MODIS instruments on the Aqua and Terra satellites on a 1 × 1 km grid. We generated a satellite‐hybrid mixed‐effects model for each year, regressing Ta measurements against land use terms, day‐specific random intercepts, and fixed and random LST slopes. We assessed model performance using 10‐fold cross‐validation at withheld stations. Across all years, the root‐mean‐square error ranged from 0.92 to 1.92 K and the *R* ^2^ ranged from .78 to .95. To demonstrate the utility of our model for health research, we evaluated the total number of days in the year 2010 when residents ≥65 years old were exposed to Ta extremes (above 30°C or below 5°C). Our model provides much needed high‐quality Ta estimates for epidemiology studies in the MCM region.

## Graphical abstract

Spatial pattern of the 95th percentiles of minimum (a) and maximum (b) temperature across days for each 1 km^2^ grid cell in the Megalopolis of Central Mexico for 2018. Temporal imputation of LST, consideration of missing data as a predictor and careful cross‐validation with detailed characterization of predictive accuracy. Application estimates population exposures to extreme temperatures for use in epidemiologic studies.

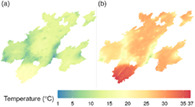

## 1 INTRODUCTION

Climate change has spurred worldwide efforts to model trends and fluctuations in near‐surface (i.e., 2 m) ambient air temperature (Ta). Recent developments in climatology and related fields have yielded valuable datasets that quantify average and extreme temperatures at local and global spatial scales and at critical time scales that are relevant for exposure science and public health (Donat *et al*., 2014; Oyler *et al*., 2015; Behnke *et al*., 2016). However, there is considerable heterogeneity in data sources, methods, and temperature products—as the process of producing Ta estimates with adequate spatial and temporal coverage is far from straightforward. Data have included in situ observations or reanalysis data from ground stations, radiosondes, satellites, and other sources (Donat *et al*., 2014; Behnke *et al*., 2016). Interpolation methods have included kriging, angular distance weighting, thin plate splines, and/or land use regression (Hofstra *et al*., 2008). The heterogeneity among different products makes them difficult to compare. Estimates are less consistent and accurate for extremes than they are for averages in Ta. Furthermore, a major limitation even among widely used datasets is that they have a relatively coarse spatial resolution that spans many kilometres (Donat *et al*., 2014; Behnke *et al*., 2016).

There are ongoing efforts in urban climate research to improve the quantification of urban meteorological phenomena, including Ta (Šećerov *et al*., 2019; Venter *et al*., 2020. Urban areas are particularly important because they contain most of the world's population; thus, more accurate estimates of the urban environment would improve research efforts linking Ta to human health risks (Venter *et al*., 2020). However, urban environments are complex and heterogeneous, and traditional meteorological monitoring stations, mostly focused on synoptic atmospheric conditions, are inadequate for capturing the spatiotemporal variability of many meteorological phenomena across urban landscapes (Meier *et al*., 2017). One direction for moving urban climatology forward is to densify urban meteorological networks by increasing the density of various sensors beyond traditional monitoring stations to include open‐source technology (Šećerov *et al*., 2019) or crowdsourced data from private monitors (Muller *et al*., 2013). There is also a trend toward integrating high‐density networks with opportunistic sensing data to improve the quantification of urban Ta (Venter *et al*., 2020). This includes remotely sensed data such as Land Surface Temperature (LST). LST is retrieved from the thermal infrared signal received by satellites and measures the thermal radiation emitted from the earth's surface as a result of the interaction between incoming solar energy and the ground, or the top of the canopy in urban and vegetated areas. LST's spatially continuous and global coverage makes it possible to examine the thermal heterogeneity of the Earth's surface and changes over time in surface temperatures (Hulley *et al*., 2019).

In epidemiology, there is a growing need for daily intracity Ta estimates (i.e., 1 × 1 km or higher resolution), as Ta effects on human health often occur at fine spatial and temporal scales (Mostofsky *et al*., 2014; Phung *et al*., 2016; Rowland *et al*., 2020; Venter *et al*., 2020. To date, many health studies only use Ta data from ground stations (Zanobetti and O'Neill, 2018). Data are often used from the airport weather stations closest to the study population or from a mix of local weather stations (Zanobetti and Schwartz, 2008; Ren *et al*., 2011; Zhang *et al*., 2017). The main problem with relying on a sparse network of ground stations or on stations located far from study populations is that it can introduce measurement bias and create exposure misclassification (Armstrong, 1998; Zeger *et al*., 2000).

Remotely sensed LST has increasingly been used to refine gridded interpolations of Ta (Oyler *et al*., 2016). LST and Ta are physically related; however, their correlation varies daily due to meteorological conditions, seasonality, soil moisture, land use, urban geometry, elevation, surface reflectance, and satellite‐surface geometry (Oyler *et al*., 2016; Shi et al., 2016a; Pelta and Chudnovsky, 2017). Researchers have in recent years incorporated satellite data and land use regression to develop more accurate estimates of Ta for epidemiology (Kloog *et al*., 2014; Ho *et al*., 2016; Shi *et al*., 2016b; Pelta and Chudnovsky, 2017; Rosenfeld *et al*., 2017; Zhang *et al*., 2017). These new models have been developed mostly for cities in northern latitudes, resulting in a geographic concentration of epidemiological research in these places (Shi *et al*., 2015; Lee *et al*., 2016; Shi *et al*., 2016a, with very few studies carried out in other regions (Xu *et al*., 2014).

In Mexico, epidemiological studies linking Ta exposure to health outcomes have mainly taken place in Mexico City and the larger Mexico City Metropolitan Area (MCMA; O'Neill *et al*., 2005; Bell *et al*., 2008; McMichael *et al*., 2008; Hurtado‐Díaz *et al*., 2019). These studies reported associations with acute mortality in different age groups, including children (0–14 years old) and the elderly (≥65 years old), who are especially vulnerable to Ta exposure due to their physiological characteristics and their dependence on caregivers to regulate their body temperature. While these studies provide valuable findings, a major shortcoming is that they assigned citywide daily average temperatures using a few weather stations with limited spatiotemporal coverage. Moreover, there is limited research outside of the MCMA and within the greater Megalopolis of Central Mexico (MCM), which includes several major metropolitan areas neighbouring the MCMA.

Ta estimates with finer spatiotemporal resolutions are needed to move beyond the citywide approach to more accurately quantify individuals' exposures. In this paper, we integrated Ta records from ground meteorological networks, satellite remote sensing LST and other spatiotemporal predictors to reconstruct daily minimum, mean, and maximum Ta for the entire MCM for the 2003–2019 time period at a 1 × 1 km spatial resolution. As a case study, we provide estimates for the total number of days in the year 2010 when MCM residents ≥65 years old were exposed to Ta extremes. Our temperature model opens up opportunities to reconstruct historical Ta exposures for any MCM resident and to explore individual‐level associations between Ta and health outcomes. These methods have yet to be applied to Mexico, a middle‐income country with unique climate zones and sociodemographic characteristics.

## 2 METHODS

### 2.1 Study area

The central region of Mexico has experienced rapid urbanization leading to the development of the MCM, which has a population of nearly 30 million inhabitants representing 25% of the country's total population. The MCM includes the MCMA in its centre and the metropolitan areas of Puebla‐Tlaxcala, Cuernavaca, Cuautla, Toluca, Pachuca, Tula, and Tulancingo. A total of 184 municipalities integrate the MCM (USAID, 2014).

Plains, mountains, and hills cover 42, 34, and 24% of the MCM territory, respectively, and its climate is influenced by the humid tropical air of the Pacific Ocean, the Caribbean Sea, and the Gulf of Mexico as well as the polar air from the North American continent. There are three main seasons in the region: cold‐dry, warm‐dry, and rainy seasons and five thermal zones (% of the total MCM territory): cold (0.7%), semicold (10%), temperate (70%), semiwarm (19%), and warm (0.3%). The annual mean temperature for the region ranges from 4 to 26°C (USAID, 2014).

**FIGURE 1:**
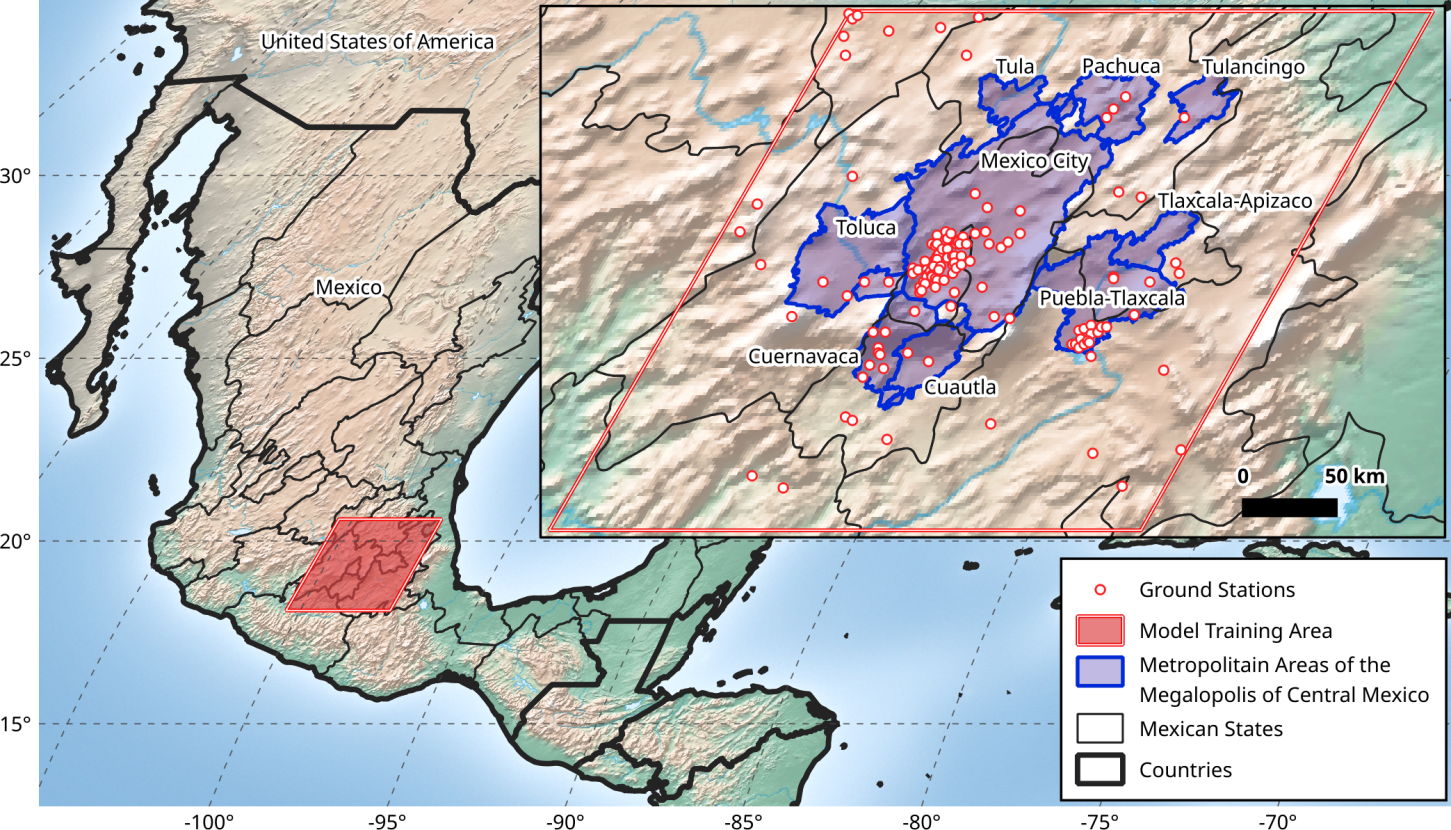
Study area showing all available ground meteorological stations (*n* = 120) used for our daily Ta predictions in the Megalopolis of Central Mexico (MCM, shown as indigo‐coloured regions) from 2003 to 2019 [Colour figure can be viewed at wileyonlinelibrary.com]

The MCM is an irregularly shaped region that is 20,686 km^2^ in area, and we used the MCM as the prediction area for which we generated and validated our Ta predictions. The longitude ranges from 99.9 to 97.8°W and the latitude ranges from 18.6 to 20.2°N (see Figure 1). To produce the study area for our Ta model, we expanded the spatial extent by 50 km outwards from each side of the bounding box of the MCM and rounded the limits to the nearest tenth of a degree of longitude or latitude. This allowed us to include additional stations that we used only as training data. The result was a rectangular region in the plate carrée projection that ranged from 100.4 to 97.4°W and 18.1 to 20.6°N. This was then reprojected to match the MODIS sinusoidal projection and divided into 101,892 total 1 × 1 km cells, and this output served as our master grid for modelling.

### 2.2 Data sources

#### 2.2.1 Meteorological ground stations

We utilized weather station records from the year 2003 through 2019 inclusive, based on data availability from the different sources of information. By request, we obtained historical records of Ta and wind speed from the Servicio Meteorológico Nacional de México (SMN) that integrated three monitoring networks: the Estaciones Meteorológicas Automáticas network (EMAs; 30 stations), the Estaciones Sinópticas Meteorológicas network (ESIMEs; 10 stations), and a network of observatories (9 stations). We also incorporated two networks for which data are publicly available on the Internet: the Sistema de Monitoreo Atmosférico de la Ciudad de México (SIMAT; 34 stations; http://www.aire.cdmx.gob.mx) and the Programa de Estaciones Meteorológicas del Bachillerato Universitario at the Universidad Nacional Autónoma de México (PEMBU‐UNAM; 13 stations; https://www.ruoa.unam.mx/pembu). Finally, we included observations from personal weather stations available from Weather Underground (70 stations; http://wunderground.com). Records for all networks are available in local time, except those from the EMAs and ESIMEs networks which are available in UTC and were converted to local time.

Given the heterogeneous sources of information in our study which included crowdsourced data, we adapted methods developed for data quality assessment applied elsewhere to make them work in our study region (Meier *et al*., 2017; Napoly *et al*., 2018; Dirksen *et al*., 2020). We discarded data that did not pass several checks. Each check, described below, is prepended with the number of observations that were deleted by the check. (a) (134) If a station had fewer than 20 observations in a given year, we dropped that station for that year. (b) (1,670) We dropped station‐days with values that were impossible (e.g., a maximum Ta that is less than the minimum Ta or a negative mean wind speed) or implausible according to climatological records (i.e., colder than −30°C, hotter than 53°C, or having a mean wind speed greater than 114 m/s). (c) (674) We dropped runs of station‐days (ignoring unobserved days) in which one of the three daily Ta values (minimum, mean, and maximum), rounded to the nearest 0.01 K, was repeatedly equal. We allowed runs of up to two equal values for the mean Ta and three equal values for the maximum or minimum Ta. These limits were chosen based on the observation that longer runs are rare except in Weather Underground. (d) (16,297) We compared observed Ta to inverse‐distance‐weighted interpolations of Ta from other stations on the same day that were no more than 30 km away and 500 m different in elevation as a “buddy check” for spatial consistency. We term the squared differences between observations and these interpolations “deviations,” and for each Ta's daily observation, we computed the 99th percentile of the deviation, excluding Weather Underground. We dropped station‐days for which a deviation exceeded this percentile, and also dropped entire stations for which 20% or more of the temperature‐days were dropped.

These checks removed 11% of Weather Underground observations, 6% of SMN‐observatories observations, and less than 5% of observations from each of the other networks. Afterwards, 402,823 observations from 166 stations remained; the above per‐network counts of stations are after data cleaning. Of these, there were 290,886 observations from 120 stations in the prediction area (i.e., the area of the MCM). We checked for possible duplicate stations (stations with different identifiers but similar locations and a substantial number of identical observations) but did not find any. A summary of the geographic location of stations from each network, number of records used in our analyses, land use/land cover, and local climate is shown in Table S1, Supporting Information. The geographic location of stations from the UNAM and REDMET networks can be found online in their websites (UNAM, https://www.ruoa.unam.mx/pembu/index.php?page=map#; REDMET, http://www.aire.cdmx.gob.mx/opendata/catalogos/cat_estacion.csv). A complete list with geographic coordinates for the EMAs and ESIMEs networks of the SMN was downloaded from their website (http://smn1.conagua.gob.mx/emas/catalogoa.html). The same information for all the networks from the SMN was received in our data request. Information on land use/land cover (Instituto Nacional de Estadística y Geografía (INEGI), 2017) and local climate (Instituto Nacional de Estadística y Geografía (INEGI), 2008) was obtained from the Instituto Nacional de Estadística y Geografía de México (INEGI) from its website. In Data S1, we included general characteristics about mounting, location, and exposure of instruments and contact information for each network, based on guidance on metadata by Aguilar *et al*. (2003), when such information was available.

#### 2.2.2 Land surface temperature

LST records were extracted from the daily daytime and night‐time MODIS LST products MOD11A1 (Terra satellite) and MYD11A1 (Aqua satellite) using the most recent reprocessing version (Collection 6). LST retrievals from both satellites were available by the first half of 2002, but we selected 2003 as the start year due to data completeness from both sensors (Tatem *et al*., 2004). The spatial resolution was 1 × 1 km, and data were available for the entire study period. Local overpass times for Terra and Aqua were around 2230 and 0130 for night‐time, and around 1030 and 1330 for daytime, respectively. MODIS LST products are derived from channels 31 (10.78–11.28 μm) and 32 (11.77–12.27 μm) in the thermal infrared band, and they are already corrected for emissivity and atmospheric effects using the split‐window algorithm. These products have been used before in similar studies due to their high spatiotemporal resolution and free availability (Benali *et al*., 2012; Kloog *et al*., 2012; 2014; Shi *et al*., 2016b; Rosenfeld *et al*., 2017).

#### 2.2.3 Land use terms

##### Vegetation

To calculate a measure of vegetation density, the monthly Normalized Difference Vegetation Index (NDVI) from both Terra and Aqua MODIS instruments were averaged (Collection 6 MOD13_A3 and MYD13_A3) at the spatial resolution of 1 × 1 km.

##### Elevation

Elevation from the Shuttle Radar Topography Mission at a 30 m spatial resolution was aggregated by applying a Gaussian filter (150 m *SD*) and extracting data to the centroids of the MODIS 1 × 1 km products.

### 2.3 Statistical methods

Aqua and Terra readings were combined to form a daily LST variable for daytime and night‐time. When one satellite's reading was missing for a given time and place, data from the other was used. When both readings were available, the average was calculated. To impute cases where both satellite readings were missing, we used a linear‐interpolation algorithm as follows. For a given day *t* and grid cell *g*, we found the closest days before and after *t* in the same year as *t*, *t*_0_, and *t*_1_, with a nonmissing value of the appropriate variable (daytime LST or night‐time LST) at *g*, and we computed the imputed value as

$$y_{\mathrm{ig}}=\left(y_{0}\left(t_{1}-t\right)+y_{1}\left(t-t_{0}\right)\right)/\left(t_{1}-t_{0}\right),$$

where *y*_*ig*_ is the LST at day *t*_*i*_ and place *g*. When no such *t*_0_ exists for the given year, *y*_1_, unaltered, is used as the imputed value instead, and likewise *y*_0_ substitutes when no *t*_1_ exists. We also imputed missing wind speed. We did this by using the wind speed from the closest station with a value for the same day (or the previous day, if no other station was available that day, or the day before that if necessary, and so on).

We calibrated daily Ta (minimum, mean, and maximum) on LST as follows: each Ta station was assigned the closest LST observation on a specific day (within each 1 × 1 km grid cell) using grid cells for which both Ta measurements and LST values (observed or imputed) were available. On each day we estimated a separate slope in the relationship between Ta and LST to capture the temporal variability in their relationship. The calibrations, fit separately per year and daily Ta outcome (minimum, mean, or maximum), were mixed‐effects regression models implemented with the R package lme4 (Bates *et al*., 2015). After calibration, we used the coefficients of the mixed‐effects model to predict Ta in those grid cells without Ta information but with LST values. A generalization of the equations for the three models (for minimum, mean, and maximum Ta) is,

$$\begin{array}{l} {\mathrm{Ta}}_{\mathrm{ij}}=\left(\alpha +\upsilon_{j}\right)+\beta_{1}\mathrm{day}\;{\mathrm{LST}}_{\mathrm{ij}}+\beta_{2}\text{night}\;{\mathrm{LST}}_{\mathrm{ij}}+\beta_{3}\text{imputed}\;\mathrm{day}\;{\mathrm{LST}}_{\mathrm{ij}}+\beta_{4}\text{imputed night}\;{\mathrm{LST}}_{\mathrm{ij}}+\\ \beta_{5}{\text{NDVI}}_{\mathrm{ij}}+\beta_{6}\sin2\pi \;{\text{time}}_{i}+\beta_{7}\cos2\pi \;{\text{time}}_{i}+\beta_{8}{\text{elevation}}_{i}+\beta_{9}\text{mean}\;{\text{wind speed}}_{\mathrm{ij}}+\beta_{10}{\text{season}}_{j}+\\ \;\beta_{11}{\text{season}}_{j}*\mathrm{day}\;{\text{surface temperature}}_{\mathrm{ij}}+\beta_{12}{\text{season}}_{j}*\text{night}\;{\text{surface temperature}}_{\mathrm{ij}}+\varepsilon_{\mathrm{ij}}, \end{array}$$

where *Ta*_*ij*_ is near‐ground (2 m) air temperature (minimum, mean, or maximum) on location *i* on day *j*; (*α + υ*_*j*_) are the fixed and random intercepts; day and night LST_*ij*_ are satellite day and satellite night LST; imputed day and night LST_*ij*_ are indicators of whether satellite day and satellite night surface temperatures are imputed; NDVI_*ij*_ is the mean NDVI of Aqua and Terra for grid cell *i* in month *j*; time_*j*_ is the time of year calculated as (day of the year^−1^)/(total days of the year^−1^); elevation_*i*_ is the mean elevation at site *i*; and mean wind speed_*ij*_ is daily mean wind speed from the nearest station. The season was defined as a categorical variable with three levels: cold‐dry for November through February, warm‐dry for March and April, and rainy for May to October. All continuous variables were centred and scaled before fitting. All analyses were conducted in R 4.0.2 (R Core Team, 2020).

#### 2.3.1 Assessment of model performance

Within each year, all available stations in the prediction area were randomly split into 10 cross‐validation folds. Inside the cross‐validation loop, models were trained with all observations (occurring in the year of interest) for stations in the training folds plus all stations outside the prediction area but within the study area (i.e., calibration folds with 90% of the data), and asked to predict the observations for the stations in the test fold (i.e., validation folds with 10% of the data). Mean wind speed for test stations was imputed as if all test stations were missing mean wind speed at all times. The primary measure of model performance was the root‐mean‐square error (RMSE) of cross‐validated predictions. Tabular summaries of model results also report the *SD* and improvement (*SD* – RMSE) of each outcome (minimum, mean, or maximum Ta) in a particular year, season, or subregion. To evaluate prediction performance more evenly throughout the study region, our summary tables include spatially weighted versions of the *SD* and RMSE, for which each day and each 16th of a longitude–latitude grid cell (splitting each degree of longitude or latitude into four equal intervals) with at least one observation is given a total weight of 1. These metrics help to evaluate performance when we consider areas with few stations (e.g., the northern parts of the MCM) to be equally important to areas with lots of stations (e.g., Mexico City proper). In addition, we estimate the degree to which our predictions capture spatial and temporal patterns by computing for each Ta outcome *y* and prediction *p*, given per‐station annual means of the outcome *M*_*y*_ and the predictions *M*_*p*_:

- *R* ^2^, the proportion of variance accounted for, as 1 − mean((*y* − *p*)^2^)/Var(*y*).
- $R_{\text{spatial}}^{2}$, the squared correlation between *M*_*y*_ and *M*_*p*_.
- $R_{\text{temporal}}^{2}$, the squared correlation between (*y* − *M*_*y*_) and (*p* − *M*_*p*_).

For summaries on the per‐station annual means ($R_{\text{spatial}}^{2}$), the sample size is the number of stations instead of station‐days (Kloog *et al*., 2014).

To verify that including Weather Underground data did not impair prediction, we conducted variations of the cross‐validation procedure in which we excluded all Weather Underground stations from testing. We computed the spatial RMSE from a cross‐validation that tests and trains in non‐Weather Underground stations, RMSE_NWU_. Then Weather Underground stations were allowed in training and RMSE_WU_ was computed in non‐Weather Underground stations and finally subtracted from RMSE_NWU_. Thus, a positive difference in RMSE_NWU_ − RMSE_WU_ means an improvement in RMSE when Weather Underground was included in training.

We constructed a learning curve in order to illustrate how our model's predictive accuracy is influenced by the size of its training data. This analysis was conducted for mean daily temperature in 2018. We selected two folds to hold out for testing, while using various subsets of the remaining eight folds for training. The test folds were chosen to have the closest unweighted RMSE under the cross‐validation as the overall RMSE for this year and dependent variable. These test folds ended up comprising 16 stations and 4,741 observations. There were seven rounds of analysis and 100 simulation replicates for each round. In each replicate of round 1, 10 stations were randomly selected, 2,500 observations were randomly selected from these 10 stations, and the model was trained on these 2,500 observations and tested on the test folds. Round 2 used 20 stations and 5,000 observations, round 3 used 30 stations and 7,500 observations, and so on up to round 7 with 70 stations and 17,500 observations.

#### 2.3.2 Estimation of at risk population

We obtained population density information at the AGEB level (equivalent to U.S. census tracts) from the 2010 Mexican Population Census that was carried out by the Instituto Nacional de Estadística y Geografía de Mexico (Instituto Nacional de Estadística y Geografía (INEGI), 2016). AGEB data are available as a polygon layer with population data for all metropolitan areas in the MCM. From these records we calculated the number of at risk days (above 30°C and below 5°C) experienced by people ≥65 years old in the MCM during the year 2010.

## 3 RESULTS

Approximately one quarter to one third of LST data were missing from both Aqua and Terra satellites and thus had to be imputed for our temperature model. The proportions of missing LST data for both satellites were similar over time (23–34% missing daytime LST and 27–37% missing night‐time LST across all years), except for a higher number of missing data in 2004 (52% daytime, 50% night‐time).

For all years and dependent variables (i.e., minimum, mean, and maximum Ta), we observed substantially lower RMSE compared with the *SD*, indicating that our model was effective in predicting temperature. Table 1 presents our model's performance in which we conducted 10‐fold cross‐validation for mean Ta. Tables S2 and S3 show cross‐validation results for minimum and maximum Ta, respectively. Across all results, the range for RMSEs was 0.9–1.9 K, with a mean of 1.5 K, whereas the range for *SD*s was 3.7–5.4 K. Values of out of sample *R* ^2^ ranged from 0.78 to 0.95 indicating good predictive abilities in our models, particularly for maximum Ta (*R* ^2^ 0.80–0.92) and mean Ta (*R* ^2^ 0.89–0.95), compared to minimum Ta (*R* ^2^ 0.78–0.87). The spatial and temporal *R* ^2^ also showed good performance with average spatial *R* ^2^ values of 0.88, 0.94, and 0.90 for minimum, mean, and maximum Ta, respectively, and average temporal *R* ^2^ values of 0.80, 0.88, and 0.85 for minimum, mean, and maximum Ta, respectively. The averaged RMSEs for minimum, mean, and maximum Ta were 1.64, 1.14, and 1.58 K, respectively, and the spatially weighted RMSEs, in which all monitored areas are equally important, were generally worse than the unweighted RMSEs (by 0.33 K on average), but still reasonably small.

**TABLE 1:** Prediction accuracy for the Megalopolis of Central Mexico: 10‐fold cross‐validation (CV) results for daily mean Ta predictions from 2003 to 2019

Year	Station‐days (*N*)	Number of stations	*SD*	RMSE	*R* ^2^	*SD*_weighted_	RMSE_weighted_	$R_{\text{spatial}}^{2}$	$R_{\text{temporal}}^{2}$
2003	9,622	32	3.94	0.92	.95	5.02	1.21	.97	.92
2004	10,453	35	3.80	1.04	.92	5.20	1.37	.93	.89
2005	11,489	36	4.16	1.09	.93	5.55	1.40	.95	.91
2006	10,882	36	3.94	1.11	.92	5.17	1.40	.95	.87
2007	9,854	39	3.95	1.04	.93	5.21	1.29	.94	.87
2008	11,430	41	4.05	1.11	.92	5.52	1.44	.96	.89
2009	13,114	48	4.13	1.21	.91	5.89	1.48	.93	.90
2010	13,980	51	4.50	1.26	.92	6.35	1.71	.95	.91
2011	14,036	46	4.25	1.16	.93	5.84	1.46	.95	.89
2012	15,161	53	3.93	1.06	.93	5.38	1.35	.96	.87
2013	17,317	59	4.21	1.14	.93	5.23	1.32	.96	.86
2014	18,685	62	4.02	1.10	.92	5.21	1.29	.96	.86
2015	20,712	69	3.92	1.09	.92	5.38	1.23	.95	.84
2016	23,716	74	4.18	1.24	.91	5.37	1.33	.94	.88
2017	23,915	80	4.15	1.30	.90	5.45	1.47	.91	.87
2018	23,558	91	3.77	1.26	.89	5.09	1.29	.90	.88
2019	29,093	99	3.68	1.22	.89	5.27	1.31	.92	.85

*Note*: SD and RMSE are in K.

**FIGURE 2:**
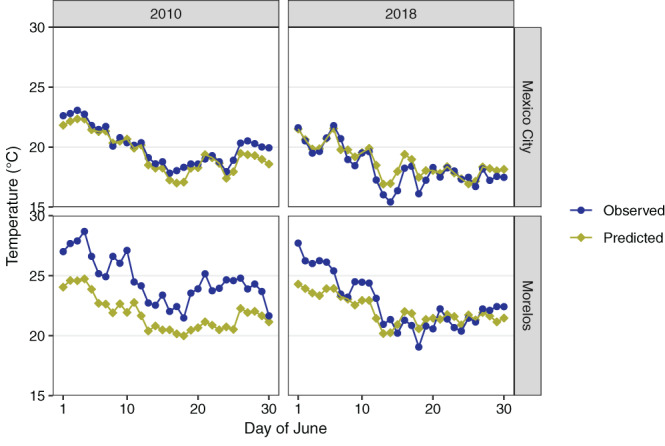
Observed and predicted Ta from CV, for station 8 (in Mexico City proper) and station 24 (in the southern region of the study area, in the state of Morelos) in two different years [Colour figure can be viewed at wileyonlinelibrary.com]

Figure 2 provides an example of observations and predictions at two different monitoring stations, in June of 2010 and 2018, showing improvement in 2018 Ta predictions compared to 2010 for the southern region of Morelos as the number of stations in training increased between these years.

**FIGURE 3:**
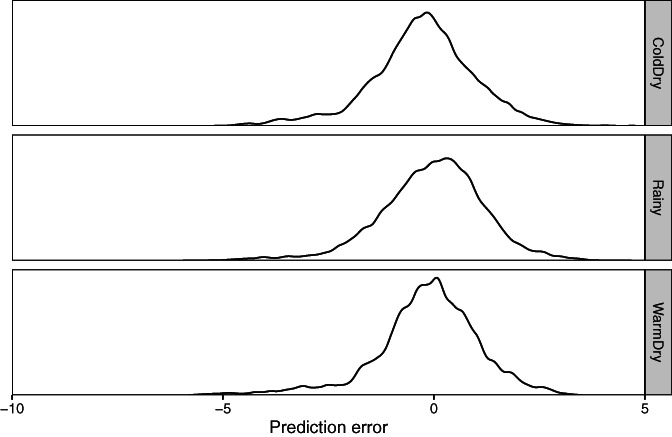
Density plots of the CV‐predicted Ta minus observed Ta in K for 2018, aggregated across stations but stratified by season

Figure 3 shows that the distribution of prediction errors in 2018 was similar by season in the MCM. Of the 23,558 predictions in the figure, 40 (1 in 589) have an error of −5 K or below. The corresponding observations are about evenly distributed by season. They come from 5 stations in the southern Valley of Mexico, 26 are from a single Weather Underground station, and 10 are from a single EMAS station.

We examined model performance by metropolitan area in order to compare how our model performed in areas with fewer stations relative to those with more stations. Table 2 summarizes cross‐validated model performance within each metropolitan area of the MCM with >5 stations for 2018. By comparing the RMSEs to the *SD*s of the Ta responses we can see how much more Ta variation is explained with our model. For results for all metropolitan areas, see Table S4. There were large differences in both *SD* and RMSE across municipalities. Still, the improvement was at least 1 K in most cases. On average, our models yield better predictions than the naïve estimate of the mean for all Ta outcomes (i.e., our predictions' measure of variation RMSE reduces the randomness in all observed Ta outcomes better than the *SD*).

**TABLE 2:** Prediction accuracy by metropolitan area in the Megalopolis of Central Mexico: 10‐fold cross‐validation (CV) results for Ta predictions for 2018

Metropolitan area	Number of stations	*N*	Temperature	*SD*	RMSE	*SD* – RMSE
Cuernavaca	7	1,717	Minimum	4.21	1.70	2.51
			Mean	4.31	1.22	3.09
			Maximum	4.41	1.93	2.47
Puebla‐Tlaxcala	16	3,553	Minimum	3.22	1.61	1.61
			Mean	2.75	1.15	1.61
			Maximum	2.99	1.57	1.42
Mexico City	65	17,092	Minimum	3.56	1.76	1.80
			Mean	3.18	1.27	1.91
			Maximum	3.60	1.45	2.14

*Note*: SD, RMSE, and *SD −* RMSE are in K.

We evaluated model performance for each ground monitoring network from which we obtained temperature records. Table 3 shows the accuracy in cross‐validated Ta predictions by type of network. The lowest RMSE values were obtained for UNAM records for minimum, mean, and maximum Ta predictions. The highest RMSE values came from ESIMEs for minimum and maximum Ta, and Weather Underground for mean Ta. One reason why UNAM had the lowest RMSEs for all Ta outcomes despite having fewer stations in 2018 compared to most networks is because UNAM stations were evenly distributed across a smaller and more climatically homogeneous area, and potentially received higher maintenance compared to the other networks.

**TABLE 3:** Prediction accuracy by ground monitoring network in the Megalopolis of Central Mexico for 2018

Ta	Network	Station‐days (*N*)	Number of stations	*SD*	RMSE	*SD* – RMSE
Minimum	EMAs	2,740	14	5.66	1.92	3.75
	ESIMEs	823	4	3.72	2.47	1.25
	SIMAT	7,787	25	3.34	1.75	1.59
	UNAM	3,187	12	2.76	1.04	1.72
	Weather Underground	8,736	35	3.66	1.89	1.77
Mean	EMAs	2,740	14	6.53	1.50	5.02
	ESIMEs	823	4	2.93	1.10	1.82
	SIMAT	7,787	25	2.77	1.04	1.73
	UNAM	3,187	12	2.47	0.72	1.74
	Weather Underground	8,736	35	3.20	1.51	1.68
Maximum	EMAs	2,740	14	7.50	2.10	5.40
	ESIMEs	823	4	3.31	2.15	1.16
	SIMAT	7,787	25	3.22	1.26	1.96
	UNAM	3,187	12	2.82	0.94	1.88
	Weather Underground	8,736	35	3.54	1.77	1.76

*Note*: SD, RMSE, and *SD* − RMSE are in K.

Model performance was compared by season type: cold‐dry, warm‐dry, and rainy. Table 4 presents the average cross‐validated prediction accuracy for each Ta outcome by season for the entire MCM from 2003 to 2019. The lowest average RMSE values were for mean Ta for all seasons. The highest RMSE values were observed for minimum and maximum Ta during the cold‐dry and rainy seasons, respectively, and for mean Ta in the cold‐dry season. The highest precision improvements were observed during the cold‐dry season for minimum Ta, and during the rainy season for mean and maximum Ta.

**TABLE 4:** Prediction accuracy by season in the Megalopolis of Central Mexico: Average *SD*, RMSE, and *SD* − RMSE for minimum, mean, and maximum Ta predictions from 2003 to 2019

Ta	Cold dry			Warm dry			Rainy		
	*SD*	RMSE	*SD* − RMSE	*SD*	RMSE	*SD* − RMSE	*SD*	RMSE	*SD* − RMSE
Minimum	3.65	1.82	1.84	3.85	1.76	2.09	3.35	1.47	1.88
Mean	3.70	1.16	2.54	4.08	1.13	2.95	3.66	1.12	2.53
Maximum	4.49	1.54	2.95	4.71	1.56	3.14	4.55	1.60	2.94

*Note*: SD, RMSE, and *SD* − RMSE are in K.

Results from our test for impaired prediction from allowing data from Weather Underground stations in training, RMSE_WU_, compared to excluding them from training, RMSE_NWU_, showed that while the mean difference of −0.04 K in RMSE_NWU_ − RMSE_WU_ favours training without Weather Underground stations, there was considerable variation in RMSE_NWU_ − RMSE_WU_ by dependent variable and year. For instance, the inclusion of Weather Underground stations improved the RMSE for minimum Ta for 7 years. Table S5 shows RMSE_NWU_ − RMSE_WU_ for all years and dependent variables in the MCM.

**FIGURE 4:**
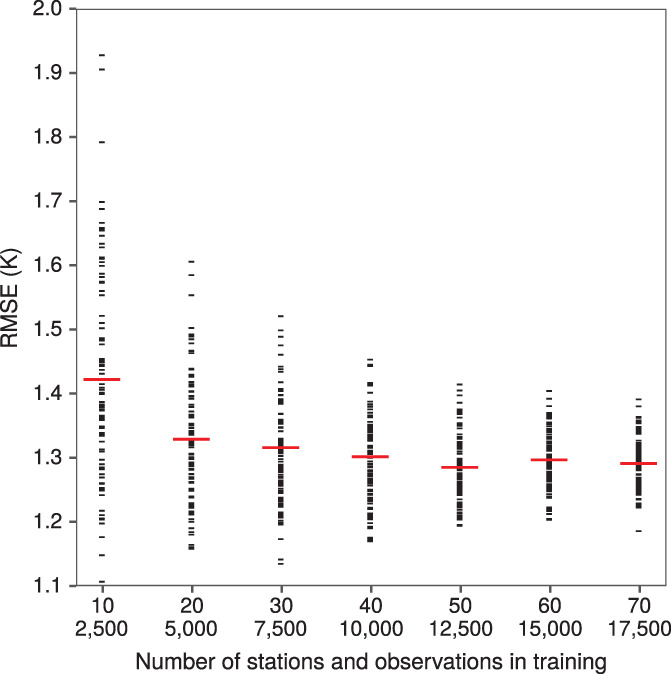
Model performance learning curve for minimum Ta in 2018 as a function of the size of its training data. Horizontal bars represent the RMSE mean [Colour figure can be viewed at wileyonlinelibrary.com]

Figure 4 shows the RMSE in two held‐out folds that can be achieved when training our model on various subsets of the remaining data. The mean RMSE across simulation replicates decreases rapidly as the training set grows from 2,500 to 7,500 observations, then levels off around 1.33 K. This example suggests that our sample is more than large enough to achieve the best accuracy possible with this model in this region.

**FIGURE 5:**
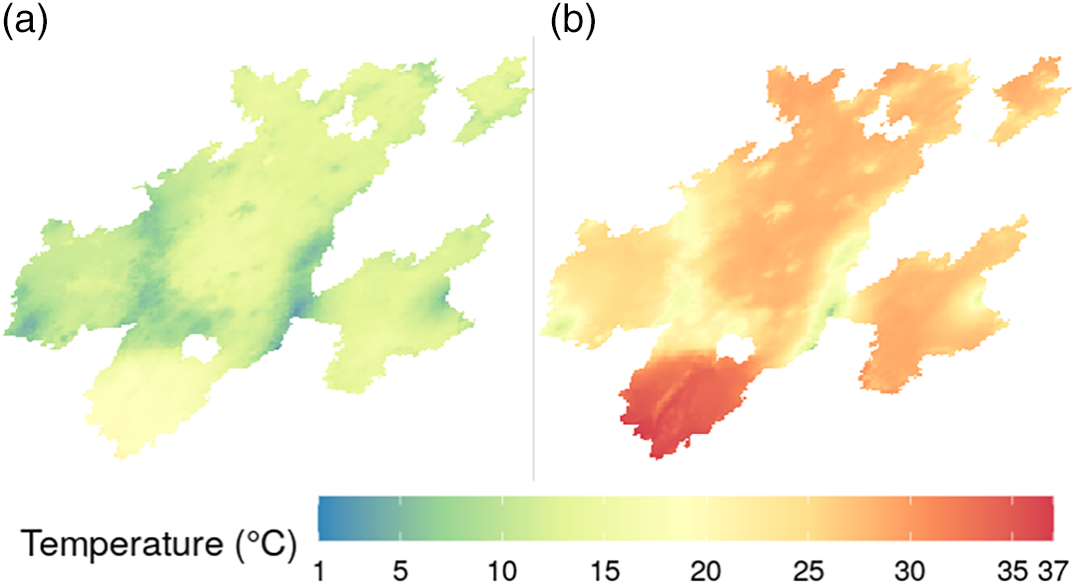
Spatial pattern of the 95th percentiles of minimum (a) and maximum (b) temperature across days for each 1 km^2^ grid cell in the Megalopolis of Central Mexico for 2018 [Colour figure can be viewed at wileyonlinelibrary.com]

Model predictions were made for each 1 × 1 km grid cell in the entire prediction area for summarization and Figure 5 shows the 95th percentile of the minimum and maximum temperatures across days for each grid cell in 2018. In general, the inhabited areas were hotter than the mountainous borders between municipalities. The southernmost region of the MCM, comprising the metropolitan areas of Cuernavaca and Cuautla, was substantially hotter than the rest of the MCM.

**FIGURE 6:**
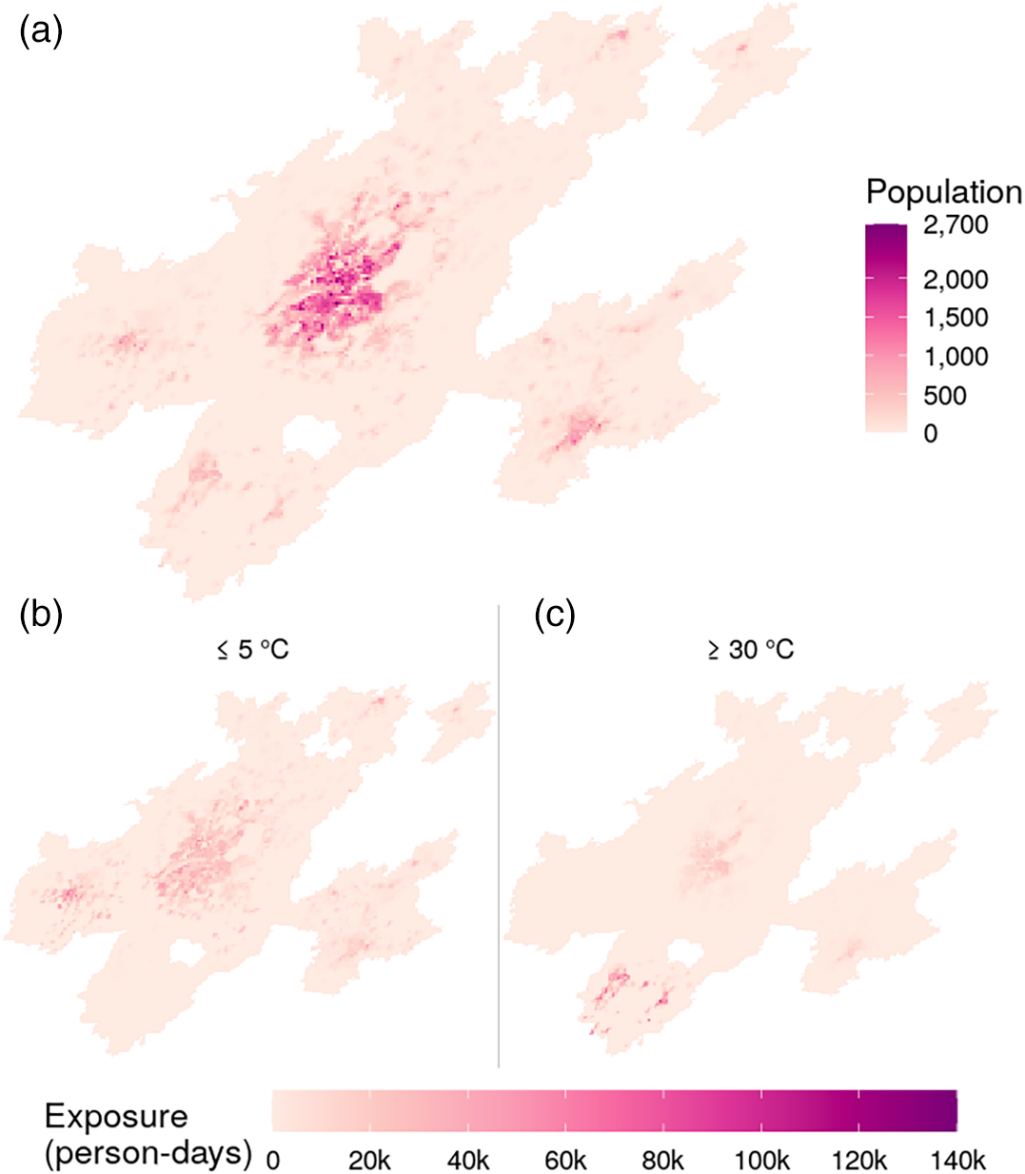
Spatial distribution of population density (a), person‐days exposure to ≤5°C (b) and ≥30°C (c) for each 1 km^2^ grid cell in the Megalopolis of Central Mexico for people ≥65 years old for 2010 [Colour figure can be viewed at wileyonlinelibrary.com]

Finally, we used population data to quantify human exposure to extreme ambient temperatures for people aged ≥65 years old in the urban AGEBs of the MCM in 2010 (Figure 6a). There were over 51 and 18 million person‐days of exposure to extreme low and high temperatures, respectively, in 2010. The highest number of person‐days of exposure to daily minimum Ta ≤5°C was concentrated in the metropolitan areas of Toluca, MCMA, Puebla‐Tlaxcala, and Pachuca (Figure 6b). As for exposure to daily maximum Ta ≥30°C, Figure 6c shows that the metropolitan areas with a higher number of person‐days above this point were Cuernavaca, Cuautla, MCMA, and Puebla‐Tlaxcala.

Raw data, processed data, predictions, and code are archived in the Zenodo open‐access digital repository (doi: http://doi.org/10.5281/zenodo.3362523).

## 4 DISCUSSION

In this paper we present the performance of our spatiotemporally resolved hybrid satellite‐based land use regression model for predicting Ta across the MCM, the most populated megacity in North America. Our daily predictions of minimum, mean, and maximum Ta are the first highly resolved 1 × 1 km air temperature estimates in the MCM. Our temperature models generated Ta predictions for an extensive region spanning several large metropolitan areas in central Mexico with diverse geographic characteristics. We addressed multiple data quality issues and incorporated many relevant predictor terms, including LST with daily varying slopes, in our models. Our high‐quality Ta predictions for the MCM have potential applications in a variety of settings such as public health, urban planning, and climatology, among others.

Consistent with results of similar studies carried out in other regions with the aim of predicting daily Ta using LST from MODIS (Benali *et al*., 2012; Kilibarda *et al*., 2014; Rosenfeld *et al*., 2017), our model predicted mean Ta more accurately than minimum or maximum Ta. Compared to previous works, the average RMSE in our models (1.64, 1.14, and 1.58 K for minimum, mean, and maximum Ta, respectively) was similar to the RMSE reported by Kloog *et al*., 2014 for the states from the northeast U.S. region (RMSE ranged from 1.43 to 2.91) for mean Ta, and to the RMSE reported by Shi et al. (2016b) (average RMSE of 1.66 K) for mean Ta in the southeast U.S. region. Results from Rosenfeld *et al*., 2017 for Israel were also similar to ours in terms of RMSE for all Ta outcomes (1.65, 0.97, 1.53 K, for minimum, mean, and maximum Ta, respectively) using LST from the MODIS instrument on the Terra satellite alone and were also similar to the RMSEs (1.65, 1.00, and 1.52 K for minimum, mean, and maximum Ta, respectively) using LST data from MODIS on the Aqua satellite alone. Previous studies agree on how often LST data are missing and that the main causes of missing LST records were related to cloud cover, weather conditions, snow, and retrieval errors. Despite the high amount of missing LST data from both Terra and Aqua satellites in 2004 (~50%) in our study region, compared to the rest of the years in the period of study, we did not observe a substantially worse performance in our models for this year (Kloog *et al*., 2014; Shi et al., 2016b; Rosenfeld *et al*., 2017). Our spatially weighted RMSEs were higher compared to those reported in previous studies which could be related to a better distribution in ground stations in other study regions (Kloog *et al*., 2014; Shi et al., 2016b; Rosenfeld *et al*., 2017). In other words, spatially weighted evaluation in our models' performance suggests worse results for subregions of the MCM with just a few ground monitoring sites such as the metropolitan areas of Toluca and Cuernavaca. In general, our model performance was similar across seasons for all daily Ta predictions.

The utility of daily Ta predictions in epidemiology from models with similar performance (i.e., RMSE) to the ones reported in our study has been demonstrated by Kloog *et al*. (2015) and Lee *et al*. (2016); both were able to assign Ta exposure with less error compared with using a closest‐monitor approach, regardless of the distance between a participant's residence and the closest ground monitor. The lower spatial resolution of Ta from ground stations increased exposure measurement error, to the extent of not finding significant associations.

As shown in Figure 6, within the MCM there are important contrasts in the spatial distribution of the at‐risk populations from exposure to extreme Ta, which justify temperature‐related epidemiological research in this region. The regions within the MCM with the greatest exposure to low temperatures correspond to the metropolitan areas of Mexico City, Pachuca, Toluca, and Puebla‐Tlaxcala. In turn, the areas with the highest concentration of hot temperatures are located in the metropolitan areas of Mexico City, Cuernavaca, Cuautla, and Puebla‐Tlaxcala. This spatial distribution of temperatures can be used for planning interventions aimed at mitigating the adverse effects from Ta on the population's health within the MCM.

One of our goals was to use the best available Ta records from ground stations in the MCM, so it is difficult to assess if our model performance is similar across the years. The underlying set of monitors that we used changed substantially with an expansion of ground monitoring from 2003 to 2019 in the MCM. A model primarily designed for long‐term climatic trends might be fit to the smaller subset of long‐running stations (Oyler *et al*., 2015). It is also possible that measurement error that is inherent to each monitoring network affected performance in our models. To address this limitation, we applied multiple criteria for quality assessment of weather records from all data sources included in our analyses. However, applying the same criteria to heterogeneous data sources to the same extent can be challenging, especially when including crowdsourced information. Unlike monitoring sites operated under international standards, private stations may not necessarily adhere to the same operation principles, with the possibility of generating data with a quality that is not suitable for research purposes (Bell *et al*., 2015). In this regard, specific quality control methods for crowdsourced data have been proposed (Droste *et al*., 2020); most of them have included statistically based steps (Chapman *et al*., 2017; Napoly *et al*., 2018) and comparisons to reference networks (Meier *et al*., 2017). Nonetheless, these methods might not be directly transferred to all locations and crowdsourced datasets, because they are only suitable for places with specific climatic characteristics and specific crowdsourced techniques, may require high‐quality reference data from dedicated urban climate observational networks, and are also dependent on records from other physical variables, which are not always available in all weather stations (Meier *et al*., 2017; Droste *et al*., 2020). Thus, replication of such methods in different settings such as the MCM may require some adaptations.

Because no reference urban‐climatic networks exist within the MCM, implementation of this approach for quality assessment of Weather Underground records in the metropolitan areas that make up the MCM is not possible. Also, the Weather Underground network in our study region includes multiple types of weather stations (see Table S1); then, quality control methods developed for specific types of weather stations such as Netatmo must be adapted to work in our study region. We implemented a quality assessment approach that was not dependent on a dedicated reference network and was not intended to be used just on a specific type of data source. In addition, we proposed and implemented a geostatistical filter to remove stations‐days considered as “deviations” by comparing observations from all networks to inverse‐distance‐weighted interpolations of temperature from neighbouring monitoring sites (excluding Weather Underground), within similar ranges of elevation. In the end, our quality assessment approach filtered out more observations from Weather Underground than from any other network, emphasizing the importance of quality assessment of crowdsourced information. We report that 26 of the 40 errors of −5 K or below in 2018 mean Ta were from a single Weather Underground station which could be caused by the inappropriate microscale siting of that respective site (e.g., next to a wall), resulting in biased measurements from that station which are small enough in magnitude to avoid flagging by our deviance criteria. Overall, the rarity of errors of this magnitude (1‐in‐589) and the robustness of model results to the exclusion of the Weather Underground network give us confidence that the trade‐offs of stringency and data availability are appropriate for our goals.

For estimating daily Ta, our model improves on previously used methods by incorporating satellite data to produce more accurate predictions for any given location within the MCM. Studies conducted in this region that aimed to estimate temperature variations beyond the spatial coverage of meteorological ground stations are scarce and not suited to generate Ta estimates with adequate spatiotemporal resolution (Carrera‐Hernández and Gaskin, 2007). The use of LST data from MODIS (alone and limited to cloud‐free conditions) in studies performed in subregions of the MCM have sought to examine seasonal variations in the urban heat island (UHI) in Mexico City using 8‐day LST averages in 2006 (Cui and de Foy, 2012) and to correlate the abundance of the dengue virus mosquito vector with 28–29‐day LST average in Puebla City (Moreno‐Madriñán *et al*., 2014). None of the above studies generated daily predictions of Ta with a potential use in intra‐urban assessment of human exposure to extreme temperatures.

Thus far, exposure assessment of Ta in epidemiological research in Mexico has relied heavily on measurements from a sparse network of ground stations that are not always located close to the populations under study (O'Neill *et al*., 2005; Bell *et al*., 2008; McMichael *et al*., 2008; Hurtado‐Díaz *et al*., 2019). The data from different monitoring networks can be challenging to work with because they are often organized heterogeneously (i.e., different file formats, temporal resolutions, time zones, differences and changes in the organization of records over time, and often coding errors), which may explain why only some and not all ground station data in Mexico have been used in previous epidemiological studies. The trade‐off is a very coarse spatial resolution, and these studies assigned daily minimum, mean, and maximum Ta exposures at spatial scales ranging from tens to a hundred kilometres.

While ground stations collect vital meteorological information, their locations constrain the utility of their data for epidemiological research particularly in urban areas. When placing ground stations, most networks follow international standards that avoid interference from large buildings, reflective surfaces, and other sources of heat or radiation (Llansó, 2003). This approach may preclude meteorological stations from being located in areas of interest for Ta‐related spatial epidemiology. For these reasons, it is important to consider the use of different quality‐controlled data sources and data fusion techniques to better reflect Ta variability within large urban areas. The inclusion of spatiotemporal covariates to predict Ta should improve on ground station data alone and would more accurately estimate the population's actual exposures (Pelta and Chudnovsky, 2017; Dirksen *et al*., 2020).

Biometeorological indices have been alternatively used to assess heat stress in epidemiology research (Basu, 2009). These biometeorological indices include apparent temperature, humidex, heat index, and net effective temperature, among others. The aim of these indices has been to reflect the actual ambient heat perceived by humans as a function of air temperature and different metrics of humidity (McGregor *et al*., 2015). However, such metrics are also more difficult to map at highly resolved spatial scales from remote sensing compared to LST. Also, the relationship between LST and biometeorological indices is complex because of the influence of factors such as wind speed and wind direction in the mass of air above the surface, land surface heat capacity, and near‐surface water available for evapotranspiration, and these data are not always available in all weather networks (Ho *et al*., 2016).

Although our temperature model offers robust predictions compared to more commonly used citywide Ta exposure estimates, our model has limitations related to its spatial and temporal resolution. It is possible that the 1 × 1 km grid cell size in our model may be too coarse of a spatial resolution for capturing biologically relevant exposures for specific types of health conditions, in steep areas with large changes in elevation, and also for exposure assessment in the different microenvironments where individuals spend most of their time. There is evidence that spatial resolutions lower than 50 m may underestimate the UHI (Sobrino *et al*., 2012), a phenomena that has been linked to different mortality and morbidity outcomes (Heaviside *et al*., 2017). Also, Ta in urban areas can change rapidly in short distances given the influence of specific city characteristics such as proximity to green areas and water bodies, surface albedo, sky view factor, and construction materials, which can induce significant variations in the actual temperature experienced by individuals within 1 × 1 km grid cells in large metropolitan areas (Schinasi *et al*., 2018). On the other hand, there is limited availability of remote sensing products with finer spatially resolved temperature data, such as the satellite data derived from USGS Landsat satellites. However, while Landsat has a higher spatial resolution, its temporal resolution is limited: once every 16 days under cloud‐free conditions (Tomlinson *et al*., 2011; Ho *et al*., 2014). Additionally, our daily temporal resolution may lead to exposure misclassification for specific acute health outcomes occurring at finer time scales. For instance, Rowland *et al*. (2020) assessed the impact of hourly temperature predictions on myocardial infarctions incidence in New York State, finding critical windows of exposure in the hours leading up to the onset of the event (Rowland *et al*., 2020). With the development of computational resources, numerical models like the Weather Research and Forecasting (WRF) model offer an alternative for capturing high‐resolution characteristics of urban climate and have been successful at simulating 2 m Ta and extreme Ta episodes such as UHIs (Yang *et al*., 2012; Jandaghian *et al*., 2018; Li *et al*., 2019). High‐resolution numerical models for studying urban climate at subkilometre scales are now possible (Jandaghian and Berardi, 2020). Recently, numerical models have been successfully employed for human exposure assessment to extreme temperatures and the projection of climate change scenarios in public health research (Ha *et al*., 2017; 2018; José *et al*., 2017; Lou *et al*., 2019). Potential limitations documented for these models are related to the available options in the WRF solver, exclusion of anthropogenic heat emissions leading to underestimation in Ta, the extensive analysis of data needed for daily long‐term simulations over years (including land use and/or urban canopy data), and comparison of area‐averaged data from simulations to observational data (point Ta observations) from reference networks (Yang *et al*., 2012; Jandaghian *et al*., 2018).

There are also potential limitations pertaining to the replicability of our methods in other places and to the comparability of our results to other studies using MODIS products to predict Ta. Previous studies in other regions have utilized different modelling techniques, have climates that are not similar to the MCM, and they have different data availability and relevance of spatial predictors such as detailed land use predictors, road density, and distance from water bodies (Kloog *et al*., 2014; Rosenfeld *et al*., 2017). Nonetheless, similar modelling approaches can generally be adopted elsewhere to improve existing Ta estimates based on limited data sources (e.g., ground stations or satellite data only).

We expect that the three different daily Ta predictions from our model can be used to improve results from prior epidemiologic research on well‐defined Ta‐related pathologies in the MCM such as cardio‐respiratory diseases and emergent vector‐borne diseases (dengue, zika, and chikungunya). They can also be used to explore new hypotheses linking Ta with birth outcomes, cardiometabolic outcomes, cognitive function, and mental health disorders (Kloog *et al*., 2015; Dai *et al*., 2016; Wallwork *et al*., 2017; Zanobetti *et al*., 2017). Our Ta predictions will facilitate investigations of the health impacts from UHI in the different metropolitan areas of the MCM and have the potential to be harnessed for early warning systems, which are increasingly important as we experience climate changes and extreme variations in temperature. Our model may assist decision‐makers in public health and meteorology in the MCM to design interventions aimed to reduce population health risks from exposure to dangerous temperature levels. While it is not a forecast system, our temperature model has the capacity to augment existing early warning systems by pinpointing more hotspots across the entire MCM and aiding the people who live there. Our exposure maps on people aged ≥65 years old exposed to extreme cold (≤5°C) and hot (≥30°C) temperatures support the idea that alert systems should consider differences in local meteorology, demographics, and urban structure to address health impacts from extreme temperatures in a megalopolitan context (McGregor *et al*., 2015).

Finally, to increase the utility of our prediction model and adhere to best practices for open and transparent research Wilson *et al*., 2017, we have archived the raw and cleaned station data, our temperature predictions, metadata, and all of the R code used in this project in a citable and open research repository (doi: http://doi.org/10.5281/zenodo.3362523). This allows others studying different parts of the world to reproduce, modify, and build on our work for the advancement of exposure science.

## 5 CONCLUSION

Evidence about the intra‐urban health effects from extreme temperatures are much needed in the Megalopolis of Central Mexico (MCM), which includes the Mexico City Metropolitan Area and several neighbouring major metropolitan areas. The aim of our study was to generate daily predictions of Ta with a spatial resolution of 1 × 1 km from 2003 to 2019, with application in public health in this region. For this we used Ta records from ground monitors and LST data from the MODIS instrument on the Terra and Aqua satellites as our main predictor of daily Ta. We calibrated LST to Ta using mixed‐effect models, land use predictors, and separate slopes for each day. Performance of our Ta models showed that daily minimum, mean, and maximum Ta can be reliably predicted using daily LST data even across the heterogeneous geography of the MCM with high accuracy. To illustrate the utility of our Ta models for public health, we calculated the number of days in the year 2010 that people ≥65 years old were at risk of exposure to extremely low or high temperatures. We estimated over 51 million person‐days of exposure to extreme cold and 18 million person‐days of exposure to extreme heat, and these exposures were concentrated in particular metropolitan areas where critical public health efforts may be most needed. Our findings reveal the potential for our daily Ta predictions to improve epidemiological research in this region.

## CONFLICT OF INTEREST

The authors declare no potential conflict of interest.

## Supporting information


**Appendix** Supporting Information

## Data Availability

Raw and processed data, including daily temperature predictions for every grid cell, as well as a research notebook with reproducible R code and instructions, are archived in Zenodo at: http://doi.org/10.5281/zenodo.3362523. Other investigators may use the raw data, cleaned meteorological data, or predictions directly, or they may rerun or modify the data‐processing and analysis code.
