# Supplementary material for: A spatiotemporal reconstruction of daily ambient temperature using satellite data in the Megalopolis of Central Mexico from 2003 to 2019
Source: Int J Climatol. 2021 Mar 18;41(8):4095–111. doi: 10.1002/joc.7060 (PMC8251982; doi:10.1002/joc.7060)
Supplement: Supplementary file 1 — Appendix Supporting Information [file JOC-41-4095-s001.docx]

**Supporting information**

**A spatiotemporal reconstruction of daily ambient temperature using satellite data in the Megalopolis of Central Mexico from 2003-2019**

**List of Contents**

Table S.1 Metadata: Location of meteorological stations in the study area after data cleaning.

Table S.2 Prediction accuracy for the Megalopolis of Central Mexico: Ten-fold cross-validation (CV) results for daily minimum Ta predictions from 2003–2019.

Table S.3 Prediction accuracy for the Megalopolis of Central Mexico: Ten-fold cross-validation (CV) results for daily maximum Ta predictions from 2003–2019.

Table S.4 Prediction accuracy: Ten-fold cross-validation (CV) results for Ta predictions in the metropolitan areas of the Megalopolis of Central Mexico for 2018.

Table S.5 Differences in RMSE_NWU_ - RMSE_WU_ for minimum, mean, and maximum Ta in the Megalopolis of Central Mexico from 2003–2019.

Supplement S1. Metadata: Weather observation station mounting, location, and exposure of instruments.

Table S.1 Metadata: Location of meteorological stations in the study area after data cleaning.

| **Network** | **Name** | **MCM region** | **Latitude** | **Longitude** | **Elevation (m)** | **Start date** | **End date** | **Number of observations** | **Land use/Land cover** | **Climate** |
| --- | --- | --- | --- | --- | --- | --- | --- | --- | --- | --- |
| EMAS | ALTZOMONI | MCMA | 19.119 | -98.655 | 3,959 | 11/1/2012 | 12/30/2019 | 2,335 | High mountain meadow | Subhumid semi-cold |
|  | APAN | TA | 19.728 | -98.466 | 2,475 | 5/13/2015 | 12/30/2019 | 1,513 | Annual rainfed agriculture | Temperate subhumid |
|  | ATLACOMULCO | TA | 19.799 | -99.877 | 2,568 | 1/1/2003 | 12/30/2019 | 5,722 | Human settlements | Temperate subhumid |
|  | CERRO CATEDRAL | Toluca | 19.542 | -99.519 | 3,385 | 1/1/2003 | 12/30/2019 | 5,533 | Oyamel-fir forest | Subhumid semi-cold |
|  | ECOGUARDAS | MCMA | 19.271 | -99.204 | 2,578 | 2/14/2008 | 12/30/2019 | 2,991 | Secondary (bushy type) vegetation of pine-oak forest | Temperate subhumid |
|  | EL CHICO | TA | 20.186 | -98.716 | 3,007 | 11/2/2012 | 12/30/2019 | 2,448 | Oyamel-fir forest | Subhumid semi-cold |
|  | ESCUELA NACIONAL DE CIENCIAS BIOLÓGICAS II, IPN. | MCMA | 19.499 | -99.145 | 2,241 | 1/1/2010 | 12/30/2019 | 2,930 | Human settlements | Temperate subhumid |
|  | ESCUELA NACIONAL DE CIENCIAS BIOLÓGICAS, IPN. | MCMA | 19.454 | -99.171 | 2,247 | 1/1/2003 | 12/30/2019 | 4,955 | Human settlements | Temperate subhumid |
|  | HUAMANTLA | TA | 19.386 | -97.966 | 2,455 | 1/1/2003 | 12/30/2019 | 5,776 | Annual rainfed agriculture | Temperate subhumid |
|  | HUAUCHINANGO | TA | 20.178 | -98.066 | 1,565 | 5/2/2008 | 12/8/2017 | 2,352 | Human settlements | Temperate humid |
|  | HUICHAPAN | TA | 20.389 | -99.664 | 2,087 | 9/1/2006 | 7/11/2019 | 4,048 | Annual rainfed agriculture | Temperate semi-dry |
|  | HUIMILPAN | TA | 20.390 | -100.283 | 2,279 | 1/1/2003 | 12/30/2019 | 5,457 | Annual rainfed agriculture | Temperate subhumid |
|  | IGUALA | TA | 18.360 | -99.524 | 766 | 11/1/2004 | 9/23/2019 | 3,836 | Human settlements | Warm subhumid |
|  | INSTITUTO MEXICANO DE TECNOLOGÍA DEL AGUA | Cuernavaca | 18.882 | -99.157 | 1,360 | 1/1/2010 | 12/30/2019 | 3,288 | Human settlements | Warm subhumid |
|  | IZUCAR DE MATAMOROS | TA | 18.617 | -98.452 | 1,310 | 1/1/2003 | 12/30/2019 | 4,974 | Semi-permanent irrigation agriculture | Warm subhumid |
|  | LA MALINCHE I | TA | 19.298 | -98.044 | 2,922 | 11/1/2012 | 12/30/2019 | 2,003 | Annual rainfed agriculture | Subhumid semi-cold |
|  | LA MALINCHE II | Puebla-Tlaxcala | 19.141 | -98.032 | 2,728 | 11/3/2012 | 12/30/2019 | 2,300 | Annual rainfed agriculture | Temperate subhumid |
|  | LAGUNAS DE ZEMPOALA | Cuernavaca | 19.053 | -99.313 | 2,846 | 11/1/2012 | 12/30/2019 | 2,099 | Pine forest | Cold |
|  | MARIPOSA MONARCA I | TA | 19.671 | -100.278 | 3,263 | 11/3/2012 | 12/25/2019 | 2,385 | Oyamel-fir forest | Subhumid semi-cold |
|  | MARIPOSA MONARCA II | TA | 19.539 | -100.290 | 3,001 | 11/14/2012 | 12/30/2019 | 2,256 | Oyamel-fir forest | Temperate subhumid |
|  | NEVADO DE TOLUCA | Toluca | 19.126 | -99.771 | 4,084 | 1/1/2003 | 12/30/2019 | 5,855 | High mountain meadow | Subhumid semi-cold |
|  | PARQUE IZTA-POPO | MCMA | 19.096 | -98.640 | 3,667 | 2/14/2008 | 12/30/2019 | 3,854 | High mountain meadow | Subhumid semi-cold |
|  | PRESA MADÍN | MCMA | 19.524 | -99.268 | 2,374 | 1/1/2003 | 11/29/2019 | 5,713 | Human-induced grassland | Temperate subhumid |
|  | SIERRA DE HUAUTLA | TA | 18.541 | -98.936 | 1,311 | 11/10/2012 | 12/30/2019 | 2,145 | Secondary (bushy type) vegetation of dry broadleaf forest | Warm subhumid |
|  | TEHUACAN | TA | 18.314 | -97.617 | 1,737 | 11/3/2012 | 12/30/2019 | 2,405 | Crassicaule shrublands | Semi-dry semi-warm |
|  | TEPOZTLAN | Cuernavaca | 18.951 | -99.079 | 1,385 | 10/22/2004 | 2/7/2019 | 4,840 | Secondary (bushy type) vegetation of dry broadleaf forest | Semi-warm subhumid |
|  | TEZONTLE | MCMA | 19.385 | -99.100 | 2,236 | 1/1/2003 | 12/30/2019 | 5,874 | Human settlements | Temperate semi-dry |
|  | TRES MARIAS | Cuernavaca | 19.051 | -99.249 | 2,832 | 1/2/2011 | 12/30/2019 | 2,142 | Annual rainfed agriculture | Temperate subhumid |
|  | UNIVERSIDAD TECNOLÓGICA DE TECAMACHALCO | TA | 18.866 | -97.722 | 2,027 | 1/1/2003 | 12/30/2019 | 5,351 | Annual rainfed agriculture | Temperate subhumid |
|  | VALLE DE BRAVO | TA | 19.376 | -100.085 | 2,514 | 11/7/2012 | 12/30/2019 | 1,859 | Annual rainfed agriculture | Temperate subhumid |
| ESIMES | CEMCAS | MCMA | 19.480 | -98.974 | 2,236 | 5/1/2014 | 5/22/2017 | 330 | Halophilic grassland | Temperate semi-dry |
|  | CUERNAVACA | Cuernavaca | 18.943 | -99.215 | 1,634 | 9/11/2009 | 12/30/2019 | 2,724 | Human settlements | Semi-warm subhumid |
|  | PACHUCA | Pachuca | 20.088 | -98.750 | 2,365 | 1/1/2013 | 10/30/2019 | 1,840 | Human settlements | Temperate semi-dry |
|  | PUEBLA | Puebla-Tlaxcala | 19.055 | -98.163 | 2,190 | 1/4/2013 | 12/30/2019 | 2,133 | Human settlements | Temperate subhumid |
|  | QUERETARO | TA | 20.563 | -100.369 | 1,902 | 1/10/2013 | 6/26/2018 | 1,543 | Human settlements | Temperate semi-dry |
|  | TACUBAYA | MCMA | 19.404 | -99.197 | 2,301 | 1/1/2006 | 12/22/2017 | 3,449 | Human settlements | Temperate subhumid |
|  | TLAXCALA | Tlaxcala-Apizaco | 19.325 | -98.247 | 2,232 | 10/9/2009 | 12/30/2019 | 3,002 | Human settlements | Temperate subhumid |
|  | TOLUCA | Toluca | 19.291 | -99.714 | 2,726 | 1/8/2013 | 12/30/2019 | 2,103 | Human settlements | Temperate subhumid |
|  | TULANCINGO | Tulancingo | 20.084 | -98.357 | 2,202 | 1/1/2006 | 3/20/2015 | 3,163 | Human settlements | Temperate semi-dry |
|  | ZACATEPEC | TA | 18.644 | -99.207 | 921 | 3/16/2013 | 12/20/2019 | 1,713 | Human settlements | Warm subhumid |
| SIMAT | ACO | MCMA | 19.636 | -98.912 | 2,259 | 7/1/2011 | 12/31/2019 | 2,859 | Human settlements | Temperate semi-dry |
|  | AJM | MCMA | 19.272 | -99.208 | 2,597 | 1/1/2015 | 12/31/2019 | 1,762 | Human settlements | Temperate subhumid |
|  | AJU | MCMA | 19.154 | -99.163 | 2,940 | 4/8/2015 | 12/31/2019 | 1,078 | Annual rainfed agriculture | Subhumid semi-cold |
|  | BJU | MCMA | 19.370 | -99.160 | 2,249 | 8/1/2015 | 12/31/2019 | 1,579 | Human settlements | Temperate subhumid |
|  | CES | MCMA | 19.335 | -99.075 | 2,247 | 1/1/2003 | 12/31/2010 | 2,663 | Human settlements | Temperate subhumid |
|  | CHO | MCMA | 19.267 | -98.886 | 2,242 | 7/1/2011 | 12/31/2019 | 2,974 | Human settlements | Temperate subhumid |
|  | CUA | MCMA | 19.365 | -99.292 | 2,690 | 1/1/2003 | 12/31/2019 | 5,431 | Human settlements | Temperate subhumid |
|  | CUT | MCMA | 19.722 | -99.199 | 2,260 | 2/21/2012 | 12/31/2019 | 2,347 | Annual and semi-permanent irrigation agriculture | Temperate subhumid |
|  | FAC | MCMA | 19.482 | -99.244 | 2,288 | 1/1/2003 | 12/31/2019 | 5,950 | Human settlements | Temperate subhumid |
|  | FAR | MCMA | 19.474 | -99.046 | 2,235 | 3/1/2019 | 12/31/2019 | 205 | Human settlements | Temperate semi-dry |
|  | GAM | MCMA | 19.483 | -99.095 | 2,239 | 12/1/2015 | 12/31/2019 | 1,443 | Human settlements | Temperate subhumid |
|  | HAN | MCMA | 19.421 | -99.084 | 2,234 | 1/1/2003 | 5/31/2006 | 1,200 | Human settlements | Temperate semi-dry |
|  | HGM | MCMA | 19.412 | -99.152 | 2,241 | 1/28/2012 | 12/31/2019 | 2,093 | Human settlements | Temperate subhumid |
|  | IMP | MCMA | 19.488 | -99.147 | 2,241 | 1/3/2008 | 3/13/2011 | 639 | Human settlements | Temperate subhumid |
|  | LAA | MCMA | 19.484 | -99.147 | 2,241 | 1/1/2016 | 12/31/2019 | 1,406 | Human settlements | Temperate subhumid |
|  | MER | MCMA | 19.425 | -99.120 | 2,238 | 1/1/2003 | 12/31/2019 | 6,054 | Human settlements | Temperate semi-dry |
|  | MGH | MCMA | 19.404 | -99.203 | 2,327 | 1/1/2015 | 12/31/2019 | 1,815 | Human settlements | Temperate subhumid |
|  | MON | MCMA | 19.460 | -98.903 | 2,246 | 1/1/2003 | 12/31/2019 | 4,759 | Semi-permanent irrigation agriculture | Temperate semi-dry |
|  | MPA | MCMA | 19.177 | -98.990 | 2,582 | 1/20/2016 | 12/31/2019 | 1,317 | Annual and permanent rainfed agriculture | Temperate subhumid |
|  | NEZ | MCMA | 19.394 | -99.028 | 2,234 | 7/1/2011 | 12/31/2019 | 2,495 | Human settlements | Temperate semi-dry |
|  | PED | MCMA | 19.325 | -99.204 | 2,346 | 1/1/2003 | 12/31/2019 | 5,542 | Human settlements | Temperate subhumid |
|  | PLA | MCMA | 19.366 | -99.200 | 2,320 | 1/1/2003 | 12/31/2010 | 2,833 | Human settlements | Temperate subhumid |
|  | SAC | MCMA | 19.346 | -99.009 | 2,286 | 3/1/2019 | 12/31/2019 | 304 | Human settlements | Temperate subhumid |
|  | SAG | MCMA | 19.533 | -99.030 | 2,236 | 1/1/2003 | 12/31/2019 | 5,431 | Human settlements | Temperate semi-dry |
|  | SFE | MCMA | 19.357 | -99.263 | 2,589 | 2/13/2012 | 12/31/2019 | 2,747 | Human settlements | Temperate subhumid |
|  | SUR | MCMA | 19.314 | -99.150 | 2,266 | 8/1/2008 | 6/24/2015 | 2,475 | Human settlements | Temperate subhumid |
|  | TAC | MCMA | 19.454 | -99.202 | 2,261 | 1/1/2003 | 12/31/2010 | 2,344 | Human settlements | Temperate subhumid |
|  | TAH | MCMA | 19.246 | -99.011 | 2,287 | 1/1/2003 | 12/31/2019 | 5,215 | Human settlements | Temperate subhumid |
|  | TLA | MCMA | 19.529 | -99.205 | 2,285 | 1/1/2003 | 12/31/2019 | 5,551 | Human settlements | Temperate subhumid |
|  | TPN | MCMA | 19.257 | -99.184 | 2,506 | 2/1/2003 | 2/22/2015 | 2,970 | Oak forest | Temperate subhumid |
|  | UAX | MCMA | 19.304 | -99.104 | 2,238 | 4/1/2015 | 12/31/2019 | 1,680 | Human settlements | Temperate subhumid |
|  | UIZ | MCMA | 19.361 | -99.074 | 2,239 | 4/1/2015 | 12/31/2019 | 1,565 | Human settlements | Temperate subhumid |
|  | VIF | MCMA | 19.658 | -99.097 | 2,245 | 1/1/2003 | 12/31/2019 | 5,620 | Human settlements | Temperate subhumid |
|  | XAL | MCMA | 19.526 | -99.082 | 2,245 | 1/1/2003 | 12/31/2019 | 5,487 | Human settlements | Temperate subhumid |
| OBSERVATORIOS | PACHUCA (OBS) | Pachuca | 20.128 | -98.748 | 2,437 | 9/12/2006 | 12/31/2016 | 635 | Human settlements | Temperate semi-dry |
|  | TULANCINGO (OBS) | Tulancingo | 20.084 | -98.357 | 2,203 | 2/3/2003 | 2/28/2010 | 749 | Human settlements | Temperate semi-dry |
|  | TOLUCA (OBS) | Toluca | 19.291 | -99.714 | 2,726 | 1/1/2003 | 7/31/2009 | 2,114 | Human settlements | Temperate subhumid |
|  | CUERNAVACA (OBS) | Cuernavaca | 18.892 | -99.233 | 1,391 | 1/1/2003 | 6/30/2010 | 1,884 | Human settlements | Semi-warm subhumid |
|  | PUEBLA (OBS) | Puebla-Tlaxcala | 19.050 | -98.167 | 2,178 | 1/1/2003 | 1/31/2010 | 2,455 | Human settlements | Temperate subhumid |
|  | UNIVERSIDAD DE PUEBLA (OBS) | Puebla-Tlaxcala | 18.996 | -98.201 | 2,138 | 1/1/2003 | 8/31/2004 | 555 | Human settlements | Temperate subhumid |
|  | QUERETARO (OBS) | TA | 20.583 | -100.400 | 1,820 | 1/1/2003 | 3/31/2010 | 1,968 | Human settlements | Semi-dry semi-warm |
|  | TLAXCALA DE XICONTECATL (OBS) | Tlaxcala-Apizaco | 19.312 | -98.244 | 2,280 | 1/1/2003 | 7/31/2010 | 1,722 | Human settlements | Temperate subhumid |
|  | TACUBAYA CENTRAL (OBS) | MCMA | 19.404 | -99.196 | 2,300 | 1/1/2003 | 12/31/2018 | 5,258 | Human settlements | Temperate subhumid |
| PEMBU-UNAM | CENTRO DE CIENCIAS DE LA ATMOSFERA | MCMA | 19.326 | -99.176 | 2,279 | 1/1/2008 | 12/31/2019 | 3,714 | Human settlements | Temperate subhumid |
|  | CCH AZCAPOTZALCO | MCMA | 19.500 | -99.204 | 2,256 | 1/28/2003 | 12/31/2019 | 5,668 | Human settlements | Temperate subhumid |
|  | CCH NAUCALPAN | MCMA | 19.474 | -99.246 | 2,337 | 7/10/2004 | 12/31/2019 | 5,480 | Human settlements | Temperate subhumid |
|  | CCH ORIENTE | MCMA | 19.384 | -99.060 | 2,238 | 1/1/2003 | 11/10/2019 | 5,384 | Human settlements | Temperate semi-dry |
|  | CCH SUR | MCMA | 19.312 | -99.199 | 2,350 | 1/1/2003 | 12/31/2019 | 5,620 | Sarcocaul shrubland | Temperate subhumid |
|  | CCH VALLEJO | MCMA | 19.484 | -99.141 | 2,241 | 1/1/2004 | 12/31/2019 | 4,817 | Human settlements | Temperate subhumid |
|  | ENP1 | MCMA | 19.271 | -99.122 | 2,242 | 1/1/2003 | 12/31/2019 | 6,070 | Human settlements | Temperate subhumid |
|  | ENP2 | MCMA | 19.384 | -99.100 | 2,236 | 1/1/2005 | 12/31/2019 | 3,592 | Human settlements | Temperate semi-dry |
|  | ENP3 | MCMA | 19.482 | -99.095 | 2,239 | 1/3/2003 | 12/31/2019 | 5,866 | Human settlements | Temperate semi-dry |
|  | ENP5 | MCMA | 19.307 | -99.133 | 2,244 | 1/1/2009 | 4/30/2019 | 2,853 | Human settlements | Temperate subhumid |
|  | ENP6 | MCMA | 19.351 | -99.156 | 2,252 | 1/1/2010 | 12/31/2019 | 2,963 | Human settlements | Temperate subhumid |
|  | ENP7 | MCMA | 19.420 | -99.127 | 2,236 | 1/1/2003 | 9/4/2019 | 5,797 | Human settlements | Temperate subhumid |
|  | ENP8 | MCMA | 19.366 | -99.195 | 2,303 | 1/1/2003 | 9/30/2019 | 5,929 | Human settlements | Temperate subhumid |
| WEATHER UNDERGROUND | 12 | TA | 19.706 | -98.336 | 2,642 | 8/27/2018 | 11/28/2019 | 341 | Annual and permanent rainfed agriculture | Temperate subhumid |
|  | 13 | TA | 20.226 | -99.049 | 2,000 | 11/8/2018 | 8/12/2019 | 256 | Annual and semi-permanent irrigation agriculture | Temperate semi-dry |
|  | 60 | MCMA | 19.398 | -99.253 | 2,492 | 4/20/2015 | 12/31/2019 | 1,573 | Human settlements | Temperate subhumid |
|  | 81 | MCMA | 19.534 | -99.237 | 2,279 | 8/16/2018 | 12/31/2019 | 492 | Human settlements | Temperate subhumid |
|  | 87 | MCMA | 19.613 | -99.098 | 2,362 | 8/1/2018 | 12/24/2019 | 90 | Human-induced grassland | Temperate subhumid |
|  | 89 | Puebla-Tlaxcala | 19.140 | -98.266 | 2,190 | 2/17/2019 | 12/31/2019 | 294 | Annual irrigation agriculture | Temperate subhumid |
|  | 121 | Cuautla | 18.916 | -98.945 | 1,449 | 4/12/2018 | 9/7/2019 | 294 | Human settlements | Semi-warm subhumid |
|  | 173 | TA | 18.471 | -97.852 | 1,862 | 10/11/2017 | 12/31/2019 | 735 | Annual rainfed agriculture | Temperate subhumid |
|  | 201 | Toluca | 19.292 | -99.502 | 2,574 | 1/1/2018 | 11/4/2019 | 478 | Annual humidity based agriculture | Temperate subhumid |
|  | 224 | MCMA | 19.170 | -99.155 | 2,861 | 2/19/2019 | 12/31/2019 | 185 | Annual rainfed agriculture | Temperate subhumid |
|  | 226 | MCMA | 19.324 | -99.229 | 2,430 | 11/14/2018 | 12/31/2019 | 257 | Human settlements | Temperate subhumid |
|  | 227 | MCMA | 19.325 | -99.123 | 2,241 | 4/21/2019 | 12/31/2019 | 226 | Human settlements | Temperate subhumid |
|  | 228 | MCMA | 19.336 | -99.134 | 2,242 | 11/12/2018 | 12/31/2019 | 366 | Human settlements | Temperate subhumid |
|  | 229 | MCMA | 19.289 | -99.186 | 2,341 | 1/10/2018 | 12/1/2019 | 626 | Human settlements | Temperate subhumid |
|  | 230 | MCMA | 19.512 | -99.133 | 2,243 | 1/13/2018 | 12/31/2019 | 703 | Human settlements | Temperate subhumid |
|  | 231 | MCMA | 19.359 | -99.205 | 2,343 | 11/4/2018 | 12/31/2019 | 412 | Human settlements | Temperate subhumid |
|  | 232 | MCMA | 19.334 | -99.279 | 2,694 | 5/30/2018 | 12/31/2019 | 576 | Human settlements | Temperate subhumid |
|  | 233 | MCMA | 19.344 | -99.197 | 2,316 | 7/26/2017 | 12/31/2019 | 739 | Human settlements | Temperate subhumid |
|  | 234 | MCMA | 19.336 | -99.198 | 2,312 | 12/11/2018 | 12/24/2019 | 314 | Human settlements | Temperate subhumid |
|  | 236 | MCMA | 19.420 | -99.211 | 2,308 | 10/8/2016 | 9/17/2019 | 1,052 | Human settlements | Temperate subhumid |
|  | 253 | MCMA | 19.422 | -99.263 | 2,422 | 1/19/2019 | 12/31/2019 | 282 | Human settlements | Temperate subhumid |
|  | 279 | Puebla-Tlaxcala | 19.068 | -98.247 | 2,125 | 4/14/2017 | 12/31/2019 | 944 | Human settlements | Temperate subhumid |
|  | 280 | Puebla-Tlaxcala | 19.080 | -98.166 | 2,229 | 5/2/2018 | 12/31/2019 | 555 | Human settlements | Temperate subhumid |
|  | 281 | Puebla-Tlaxcala | 18.993 | -98.245 | 2,118 | 5/22/2018 | 12/31/2019 | 428 | Human settlements | Temperate subhumid |
|  | 282 | Puebla-Tlaxcala | 19.065 | -98.264 | 2,137 | 6/7/2018 | 12/31/2019 | 565 | Annual and semi-permanent irrigation agriculture | Temperate subhumid |
|  | 283 | Puebla-Tlaxcala | 19.085 | -98.219 | 2,158 | 6/7/2018 | 12/31/2019 | 560 | Human settlements | Temperate subhumid |
|  | 284 | Puebla-Tlaxcala | 19.044 | -98.241 | 2,119 | 4/9/2019 | 11/14/2019 | 177 | Human settlements | Temperate subhumid |
|  | 332 | TA | 20.571 | -99.724 | 1,791 | 8/12/2018 | 12/31/2019 | 387 | Annual rainfed agriculture | Semi-dry semi-warm |
|  | 333 | TA | 18.939 | -97.919 | 2,200 | 10/28/2018 | 12/31/2019 | 290 | Annual and semi-permanent irrigation agriculture | Temperate subhumid |
|  | 349 | TA | 19.152 | -100.126 | 1,999 | 1/4/2019 | 12/18/2019 | 277 | Pine forest | Temperate subhumid |
|  | 444 | MCMA | 19.247 | -99.187 | 2,631 | 2/19/2010 | 12/31/2019 | 3,444 | Oak forest | Temperate subhumid |
|  | 446 | MCMA | 19.429 | -99.200 | 2,276 | 2/16/2015 | 12/31/2019 | 1,722 | Human settlements | Temperate subhumid |
|  | 447 | MCMA | 19.329 | -99.206 | 2,343 | 1/1/2016 | 12/31/2019 | 1,298 | Human settlements | Temperate subhumid |
|  | 450 | MCMA | 19.127 | -98.741 | 2,542 | 1/1/2016 | 12/31/2019 | 913 | Annual rainfed agriculture | Temperate subhumid |
|  | 451 | TA | 19.184 | -100.127 | 1,804 | 9/8/2014 | 12/31/2019 | 1,291 | Human settlements | Temperate subhumid |
|  | 452 | TA | 19.184 | -100.127 | 1,802 | 2/27/2015 | 6/6/2019 | 1,277 | Human settlements | Temperate subhumid |
|  | 453 | MCMA | 19.531 | -98.847 | 2,272 | 2/24/2012 | 12/31/2019 | 2,679 | Annual and semi-permanent irrigation agriculture | Temperate subhumid |
|  | 461 | TA | 18.308 | -99.332 | 956 | 9/14/2014 | 12/20/2019 | 1,810 | Human settlements | Warm subhumid |
|  | 462 | TA | 18.345 | -99.538 | 744 | 9/14/2008 | 12/20/2019 | 1,752 | Human settlements | Warm subhumid |
|  | 475 | Cuernavaca | 18.978 | -99.243 | 1,844 | 1/1/2016 | 12/31/2019 | 1,384 | Human settlements | Temperate subhumid |
|  | 476 | Cuernavaca | 18.952 | -99.229 | 1,660 | 6/20/2016 | 12/31/2019 | 936 | Human settlements | Semi-warm subhumid |
|  | 477 | Cuernavaca | 18.839 | -99.234 | 1,243 | 4/5/2008 | 12/31/2019 | 1,987 | Human settlements | Warm subhumid |
|  | 478 | TA | 18.625 | -99.168 | 906 | 7/21/2015 | 6/26/2019 | 698 | Human settlements | Warm subhumid |
|  | 494 | TA | 19.496 | -97.594 | 2,352 | 3/30/2015 | 12/31/2019 | 1,646 | Annual rainfed agriculture | Temperate subhumid |
|  | 495 | Puebla-Tlaxcala | 18.978 | -98.221 | 2,104 | 9/5/2013 | 12/31/2019 | 1,266 | Human settlements | Temperate subhumid |
|  | 496 | Puebla-Tlaxcala | 19.013 | -98.194 | 2,139 | 4/30/2013 | 12/31/2019 | 2,107 | Human settlements | Temperate subhumid |
|  | 497 | Puebla-Tlaxcala | 19.076 | -98.140 | 2,255 | 9/4/2013 | 12/31/2019 | 1,486 | Human settlements | Temperate subhumid |
|  | 498 | Puebla-Tlaxcala | 19.003 | -98.184 | 2,137 | 10/5/2017 | 11/17/2019 | 668 | Human settlements | Temperate subhumid |
|  | 499 | Puebla-Tlaxcala | 19.044 | -98.196 | 2,152 | 12/4/2008 | 12/5/2019 | 3,709 | Human settlements | Temperate subhumid |
|  | 500 | TA | 18.490 | -97.416 | 1,674 | 5/17/2012 | 12/31/2019 | 2,502 | Annual rainfed agriculture | Semi-dry semi-warm |
|  | 505 | TA | 20.479 | -100.356 | 2,005 | 8/17/2006 | 12/31/2019 | 3,981 | Annual irrigation agriculture | Temperate semi-dry |
|  | 506 | TA | 20.370 | -100.270 | 2,287 | 7/21/2006 | 12/31/2019 | 4,001 | Human-induced grassland | Temperate subhumid |
|  | 507 | TA | 20.503 | -100.144 | 1,920 | 6/29/2006 | 12/31/2019 | 4,483 | Human settlements | Temperate semi-dry |
|  | 509 | TA | 20.578 | -100.350 | 1,999 | 7/14/2006 | 12/31/2019 | 4,004 | Secondary (tree type) vegetation of dry broadleaf forest | Temperate semi-dry |
|  | 510 | TA | 20.534 | -100.212 | 1,925 | 10/4/2006 | 12/31/2019 | 3,774 | Annual and semi-permanent irrigation agriculture | Temperate semi-dry |
|  | 515 | TA | 20.384 | -99.988 | 1,945 | 4/19/2007 | 12/31/2019 | 3,505 | Human settlements | Temperate semi-dry |
|  | 516 | TA | 20.370 | -100.002 | 1,940 | 4/19/2007 | 12/26/2019 | 3,733 | Human settlements | Temperate semi-dry |
|  | 562 | Toluca | 19.227 | -99.551 | 2,583 | 7/30/2014 | 12/26/2019 | 522 | Annual rainfed agriculture | Temperate subhumid |
|  | 563 | TA | 19.341 | -97.920 | 2,465 | 11/22/2012 | 12/31/2019 | 2,330 | Human settlements | Temperate subhumid |
|  | 805 | TA | 19.464 | -97.688 | 2,393 | 6/6/2019 | 12/26/2019 | 126 | Human settlements | Temperate subhumid |
|  | 817 | TA | 20.565 | -100.242 | 1,915 | 5/18/2019 | 12/31/2019 | 197 | Annual and semi-permanent irrigation agriculture | Temperate semi-dry |
|  | 832 | TA | 18.592 | -99.260 | 970 | 6/4/2019 | 12/31/2019 | 191 | Annual rainfed agriculture | Warm subhumid |
|  | 844 | MCMA | 19.323 | -99.201 | 2,342 | 9/25/2017 | 12/31/2019 | 711 | Human settlements | Temperate subhumid |
|  | 862 | MCMA | 19.476 | -99.268 | 2,419 | 3/12/2017 | 12/15/2019 | 645 | Human settlements | Temperate subhumid |
|  | 864 | Puebla-Tlaxcala | 19.128 | -98.220 | 2,188 | 1/30/2019 | 7/14/2019 | 80 | Human settlements | Temperate subhumid |
|  | 876 | MCMA | 19.405 | -99.334 | 2,661 | 5/22/2019 | 10/25/2019 | 141 | Human settlements | Temperate subhumid |
|  | 886 | TA | 18.275 | -98.265 | 1,133 | 5/10/2019 | 12/31/2019 | 211 | Annual rainfed agriculture | Warm subhumid |
|  | 906 | Puebla-Tlaxcala | 19.022 | -98.232 | 2,105 | 7/5/2017 | 12/2/2019 | 589 | Human settlements | Temperate subhumid |
|  | 910 | TA | 20.188 | -100.142 | 2,623 | 6/20/2006 | 12/31/2019 | 3,929 | Human settlements | Temperate subhumid |
|  | 914 | TA | 20.520 | -99.885 | 1,878 | 6/29/2006 | 12/31/2019 | 4,456 | Human settlements | Temperate semi-dry |

Table S.2 Prediction accuracy for the Megalopolis of Central Mexico: Ten-fold cross-validation (CV) results for daily minimum Ta predictions from 2003–2019.

| **Year** | **Station-Days (N)** | **Number of Stations** | **S.D.** | **RMSE** | **R^2^** | **S.D._weighted_** | **RMSE_weighted_** | **R^2^spatial** | **R^2^ temporal** |
| --- | --- | --- | --- | --- | --- | --- | --- | --- | --- |
| 2003 | 9622 | 32 | 4.08 | 1.46 | 0.87 | 4.85 | 1.93 | 0.90 | 0.85 |
| 2004 | 10453 | 35 | 3.92 | 1.53 | 0.85 | 4.94 | 2.03 | 0.82 | 0.84 |
| 2005 | 11489 | 36 | 4.02 | 1.61 | 0.84 | 5.10 | 2.16 | 0.87 | 0.82 |
| 2006 | 10882 | 36 | 4.04 | 1.52 | 0.86 | 4.94 | 1.96 | 0.87 | 0.84 |
| 2007 | 9854 | 39 | 3.90 | 1.50 | 0.85 | 4.85 | 1.86 | 0.88 | 0.79 |
| 2008 | 11430 | 41 | 4.12 | 1.55 | 0.86 | 5.13 | 2.00 | 0.94 | 0.82 |
| 2009 | 13114 | 48 | 4.02 | 1.58 | 0.85 | 5.33 | 2.05 | 0.88 | 0.80 |
| 2010 | 13980 | 51 | 4.53 | 1.63 | 0.87 | 5.67 | 2.11 | 0.90 | 0.86 |
| 2011 | 14036 | 46 | 4.25 | 1.60 | 0.86 | 5.28 | 1.97 | 0.90 | 0.83 |
| 2012 | 15161 | 53 | 3.90 | 1.59 | 0.83 | 4.91 | 1.96 | 0.91 | 0.77 |
| 2013 | 17317 | 59 | 4.16 | 1.71 | 0.83 | 4.89 | 1.98 | 0.90 | 0.75 |
| 2014 | 18685 | 62 | 4.21 | 1.63 | 0.85 | 5.14 | 1.97 | 0.90 | 0.79 |
| 2015 | 20712 | 69 | 3.96 | 1.63 | 0.83 | 5.16 | 1.84 | 0.87 | 0.75 |
| 2016 | 23716 | 74 | 4.25 | 1.87 | 0.81 | 5.26 | 2.05 | 0.85 | 0.78 |
| 2017 | 23915 | 80 | 4.54 | 1.92 | 0.82 | 5.60 | 2.26 | 0.84 | 0.82 |
| 2018 | 23558 | 91 | 3.96 | 1.77 | 0.80 | 5.01 | 1.90 | 0.85 | 0.80 |
| 2019 | 29093 | 99 | 3.88 | 1.83 | 0.78 | 5.15 | 2.02 | 0.81 | 0.76 |

SD and RMSE in K.

Table S.3 Prediction accuracy for the Megalopolis of Central Mexico: Ten-fold cross-validation (CV) results for daily maximum Ta predictions from 2003–2019.

| **Year** | **Station-Days (N)** | **Number of Stations** | **S.D.** | **RMSE** | **R^2^** | **S.D._weighted_** | **RMSE_weighted_** | **R^2^spatial** | **R^2^ temporal** |
| --- | --- | --- | --- | --- | --- | --- | --- | --- | --- |
| 2003 | 9622 | 32 | 4.64 | 1.35 | 0.92 | 6.04 | 1.82 | 0.96 | 0.88 |
| 2004 | 10453 | 35 | 4.58 | 1.35 | 0.91 | 6.33 | 1.76 | 0.94 | 0.86 |
| 2005 | 11489 | 36 | 4.96 | 1.48 | 0.91 | 6.67 | 1.87 | 0.94 | 0.87 |
| 2006 | 10882 | 36 | 4.76 | 1.47 | 0.90 | 6.29 | 1.84 | 0.94 | 0.84 |
| 2007 | 9854 | 39 | 4.82 | 1.47 | 0.91 | 6.39 | 1.88 | 0.93 | 0.84 |
| 2008 | 11430 | 41 | 4.85 | 1.59 | 0.89 | 6.78 | 1.96 | 0.88 | 0.85 |
| 2009 | 13114 | 48 | 4.99 | 1.69 | 0.88 | 7.20 | 2.12 | 0.85 | 0.87 |
| 2010 | 13980 | 51 | 5.36 | 1.67 | 0.90 | 7.73 | 2.30 | 0.93 | 0.88 |
| 2011 | 14036 | 46 | 5.03 | 1.59 | 0.90 | 7.16 | 2.05 | 0.92 | 0.87 |
| 2012 | 15161 | 53 | 4.71 | 1.46 | 0.90 | 6.57 | 1.83 | 0.91 | 0.87 |
| 2013 | 17317 | 59 | 4.97 | 1.69 | 0.88 | 6.35 | 2.12 | 0.89 | 0.85 |
| 2014 | 18685 | 62 | 4.66 | 1.65 | 0.87 | 6.13 | 2.03 | 0.89 | 0.83 |
| 2015 | 20712 | 69 | 4.60 | 1.60 | 0.88 | 6.28 | 2.01 | 0.89 | 0.82 |
| 2016 | 23716 | 74 | 4.82 | 1.64 | 0.88 | 6.15 | 2.05 | 0.89 | 0.87 |
| 2017 | 23915 | 80 | 4.47 | 1.66 | 0.86 | 6.00 | 2.04 | 0.87 | 0.82 |
| 2018 | 23558 | 91 | 4.18 | 1.58 | 0.86 | 5.66 | 1.82 | 0.85 | 0.86 |
| 2019 | 29093 | 99 | 4.17 | 1.86 | 0.80 | 5.96 | 2.18 | 0.78 | 0.81 |

SD and RMSE in K.

Table S.4 Prediction accuracy: Ten-fold cross-validation (CV) results for Ta predictions in the metropolitan areas of the Megalopolis of Central Mexico for 2018.

| **Temperature** | **Metropolitan area** | **N** | **Number of stations** | **S.D.** | **RMSE** | **S.D. ─ RMSE** |
| --- | --- | --- | --- | --- | --- | --- |
| Minimum | Cuautla | 196 | 1 | 1.41 | 1.51 | -0.09 |
|  | Cuernavaca | 1717 | 7 | 4.21 | 1.7 | 2.51 |
|  | Pachuca | 132 | 1 | 3.48 | 2.14 | 1.34 |
|  | Puebla-Tlaxcala | 3415 | 13 | 3.22 | 1.61 | 1.61 |
|  | Tlaxcala-Apizaco | 224 | 1 | 2.94 | 2.54 | 0.4 |
|  | Toluca | 1015 | 4 | 3.44 | 2.34 | 1.1 |
|  | Mexico City | 16859 | 64 | 3.56 | 1.76 | 1.8 |
| Mean | Cuautla | 196 | 1 | 1.59 | 1.04 | 0.55 |
|  | Cuernavaca | 1717 | 7 | 4.31 | 1.22 | 3.09 |
|  | Pachuca | 132 | 1 | 3.28 | 1.1 | 2.18 |
|  | Puebla-Tlaxcala | 3415 | 13 | 2.75 | 1.15 | 1.61 |
|  | Tlaxcala-Apizaco | 224 | 1 | 2.16 | 1.11 | 1.06 |
|  | Toluca | 1015 | 4 | 4.59 | 1.59 | 2.99 |
|  | Mexico City | 16859 | 64 | 3.18 | 1.27 | 1.91 |
| Maximum | Cuautla | 196 | 1 | 2.54 | 1.22 | 1.32 |
|  | Cuernavaca | 1717 | 7 | 4.41 | 1.93 | 2.47 |
|  | Pachuca | 132 | 1 | 4.29 | 3.42 | 0.87 |
|  | Puebla-Tlaxcala | 3415 | 13 | 2.99 | 1.57 | 1.42 |
|  | Tlaxcala-Apizaco | 224 | 1 | 2.71 | 1.73 | 0.98 |
|  | Toluca | 1015 | 4 | 6.5 | 2.49 | 4.01 |
|  | Mexico City | 16859 | 64 | 3.6 | 1.45 | 2.14 |

SD, RMSE, and SD-RMSE in K.

Table S.5 Differences in RMSE_NWU_ - RMSE_WU_ for minimum, mean, and maximum Ta in the Megalopolis of Central Mexico from 2003–2019.

| **Dependent Variable** | **Year** | **N** | **RMSE_NWU_** | **RMSEN_WU_** | **RMSE_NWU_ - RMSEN_WU_** |
| --- | --- | --- | --- | --- | --- |
| Minimum Ta | 2003 | 9622 | 1.93 | 1.93 | 0.00 |
|  | 2004 | 10453 | 2.03 | 2.03 | 0.00 |
|  | 2005 | 11489 | 2.16 | 2.16 | 0.00 |
|  | 2006 | 10882 | 1.97 | 1.96 | 0.01 |
|  | 2007 | 9854 | 1.85 | 1.86 | -0.01 |
|  | 2008 | 11378 | 1.98 | 2.00 | -0.01 |
|  | 2009 | 12743 | 2.07 | 2.07 | 0.00 |
|  | 2010 | 13357 | 2.07 | 2.16 | -0.09 |
|  | 2011 | 13376 | 2.00 | 1.98 | 0.02 |
|  | 2012 | 14074 | 1.97 | 1.94 | 0.03 |
|  | 2013 | 15622 | 1.98 | 1.96 | 0.02 |
|  | 2014 | 16907 | 1.99 | 1.97 | 0.03 |
|  | 2015 | 18598 | 1.85 | 1.84 | 0.00 |
|  | 2016 | 19899 | 2.09 | 2.08 | 0.02 |
|  | 2017 | 18459 | 2.28 | 2.31 | -0.03 |
|  | 2018 | 14822 | 1.90 | 1.95 | -0.04 |
|  | 2019 | 17058 | 2.04 | 2.05 | -0.01 |
| Mean Ta | 2003 | 9622 | 1.21 | 1.21 | 0.00 |
|  | 2004 | 10453 | 1.37 | 1.37 | 0.00 |
|  | 2005 | 11489 | 1.40 | 1.40 | 0.00 |
|  | 2006 | 10882 | 1.40 | 1.40 | 0.00 |
|  | 2007 | 9854 | 1.30 | 1.29 | 0.01 |
|  | 2008 | 11378 | 1.39 | 1.44 | -0.05 |
|  | 2009 | 12743 | 1.46 | 1.48 | -0.02 |
|  | 2010 | 13357 | 1.41 | 1.74 | -0.33 |
|  | 2011 | 13376 | 1.47 | 1.46 | 0.01 |
|  | 2012 | 14074 | 1.39 | 1.38 | 0.02 |
|  | 2013 | 15622 | 1.34 | 1.34 | 0.01 |
|  | 2014 | 16907 | 1.30 | 1.29 | 0.01 |
|  | 2015 | 18598 | 1.19 | 1.21 | -0.02 |
|  | 2016 | 19899 | 1.26 | 1.29 | -0.03 |
|  | 2017 | 18459 | 1.37 | 1.43 | -0.06 |
|  | 2018 | 14822 | 1.10 | 1.24 | -0.14 |
|  | 2019 | 17058 | 1.13 | 1.29 | -0.16 |
| Maximum Ta | 2003 | 9622 | 1.82 | 1.82 | 0.00 |
|  | 2004 | 10453 | 1.77 | 1.77 | 0.00 |
|  | 2005 | 11489 | 1.88 | 1.88 | 0.00 |
|  | 2006 | 10882 | 1.84 | 1.84 | 0.00 |
|  | 2007 | 9854 | 1.87 | 1.88 | -0.01 |
|  | 2008 | 11378 | 1.80 | 1.94 | -0.14 |
|  | 2009 | 12743 | 2.03 | 2.10 | -0.07 |
|  | 2010 | 13357 | 1.79 | 2.30 | -0.51 |
|  | 2011 | 13376 | 1.97 | 2.03 | -0.06 |
|  | 2012 | 14074 | 1.81 | 1.83 | -0.02 |
|  | 2013 | 15622 | 2.10 | 2.15 | -0.04 |
|  | 2014 | 16907 | 2.05 | 2.07 | -0.02 |
|  | 2015 | 18598 | 1.99 | 2.00 | -0.01 |
|  | 2016 | 19899 | 2.01 | 2.03 | -0.02 |
|  | 2017 | 18459 | 1.98 | 2.01 | -0.04 |
|  | 2018 | 14822 | 1.73 | 1.81 | -0.08 |
|  | 2019 | 17058 | 1.95 | 2.13 | -0.17 |

RMSE and SD-RMSE in K.

Positive RMSE_NWU_ - RMSE_WU_ means an improvement in RMSE when Weather Underground stations were included in training.

Supplement S.1. Metadata: Weather observation station mounting, location, and exposure of instruments.

Although our research project entails a secondary data analysis without primary data collection, we have also aggregated and translated metadata that we were able to gather from the meteorological observation networks with a focus on temperature and wind speed instruments. For each network, we list the name of the document, link to the metadata source, the date that we last accessed these materials, and translate the relevant details. It is important to note that these metadata may become out of date as the underlying information may change. We include links to the Internet Archive for broken hyperlinks that may be the result of changing governmental websites.

1. The Estaciones Meteorológicas Automáticas network (EMAs), Estaciones Sinópticas Meteorológicas network (ESIMEs), and Observatorios network of the Sistema Meteorológico Nacional (SMN)

| Document Name (Original):  NORMA MEXICANA NMX-AA-166/1-SCFI-2013  ESTACIONES CLIMATOLÓGICAS E HIDROLÓGICAS - PARTE 1: ESPECIFICACIONES TÉCNICAS QUE DEBEN CUMPLIR LOS MATERIALES E INSTRUMENTOS DE MEDICIÓN DE LAS ESTACIONES METEOROLÓGICAS AUTOMÁTICAS Y CONVENCIONALES |
| --- |
| Document Name (Translated):  MEXICAN STANDARD NMX-AA-166/1-SCFI-2013  WEATHER, CLIMATOLOGICAL, AND HYDROLOGICAL STATIONS. PART 1: TECHNICAL SPECIFICATIONS TO BE ACCOMPLISHED BY THE MATERIALS AND MEASURING INSTRUMENTS FOR THE AUTOMATIC AND CONVENTIONAL METEOROLOGICAL STATIONS |
| Data Source: <https://www.gob.mx/cms/uploads/attachment/file/166835/nmx-aa-166-1-scfi-2013_1_.pdf> |
| Last Access Date: 05/27/2020 |
| Document Name (Original):  NORMA MEXICANA NMX-AA-166/2-SCFI-2015 ESTACIONES METEOROLÓGICAS, CLIMATOLÓGICAS E HIDROLÓGICAS. PARTE 2: ESPECIFICACIONES TÉCNICAS QUE DEBEN CUMPLIR EL EMPLAZAMIENTO Y EXPOSICIÓN DE LOS INSTRUMENTOS DE MEDICIÓN DE LAS ESTACIONES METEOROLÓGICAS AUTOMÁTICAS Y CONVENCIONALES. |
| Document Name (Translated):  WEATHER STATIONS, CLIMATOLOGICAL AND HYDROLOGICAL. PART 2: TECHNICAL SPECIFICATIONS TO BE ACCOMPLISHED BY THE SITING AND MEASURING INSTRUMENTS FOR THE AUTOMATIC AND CONVENTIONAL METEOROLOGICAL STATIONS |
| Data source: <https://www.gob.mx/cms/uploads/attachment/file/166838/nmx-aa-166-2-scfi-2015.pdf> |
| Last Access Date: 05/27/2020 |

These networks base their siting and operation protocols for meteorological stations, as well as the exposure of the measuring instruments on the Mexican standards: NMX-AA-166/1-SCFI-2013 and NMX-AA-166/2-SCFI-2015 for “Weather, climatological and hydrological stations. Technical specifications to be accomplished by the materials and measuring instruments for the automatic and conventional meteorological stations in Mexico”. These standards are in line with international guidelines.

- 1. Technical specifications of sensors for temperature and wind speed

*Observatorios (Conventional Synoptic Surface Stations type)*

Temperature

- Instrument: Room thermometer
- Technical specifications: Mercury Type
- Scale : Degree Celsius °C
- Range: - 30 to + 60 °C
- Accuracy: ± 0.1 °C
- Resolution: 0.2 °C

Wind speed

- Instrument: Anemometer
- Technical specifications: Cup or digital
- Material: Stainless steel or anodized aluminum cups with a corrosion resistant protective coating.
- Measurement range: 0 to 65 m/s = 234 km/h
- Accuracy: ± 2%
- Resolution: 0.27 m/s
- Response threshold: 0.7 m/s = 2.52 km/h
- Scale: m/s, km/h
- Wind speed sensor should work perfectly at an ambient temperature ranging from - 40 °C to 60 °C

*Automatic Meteorological Stations (EMAs) and Synoptic Meteorological Stations (ESIMEs)*

Temperature sensor

- Measurement range: - 40 °C to 60 °C
- Accuracy: +/- 0.2ºC at 20 °C
- Resolution: 0.1 °C

Wind speed sensor

- Measurement range: 0 to 75 m / s = 270 km / h
- Accuracy: ± 0.2m/s
- Resolution: 0.01 m/s
  1. Location and exposure of instruments
- Stations are located at sites covered with native grass that is kept short.
- In a radius of 50 m around each station the natural vegetation should not exceed 8 m in height.
- Stations are free of natural or artificial obstacles (trees, buildings, etc.), within a distance equivalent to ten times its height.

*Temperature instruments*

- Instruments are placed between 1.25 m and 2.00 m above ground level, and are also placed inside a stand for protection.
- The measurement points are located more than 100 m from a heat source or reflective surface (buildings, concrete surfaces, parking lots, etc.), more than 100 m from a body of water (unless it is important in the region) and far from any shadow projection when the sun position exceeds 5º.

*Wind speed instruments*

- Instruments are placed at a height of 10 m above the ground surface and on flat and open terrain free of obstacles.
- Instruments are placed on a metal mast or tower, either at the top of it or on one side of the tower on a metal arm up to a distance of at least twice the diameter/diagonal of the tower, extended towards outside in the direction of the prevailing wind.
  1. Contact information

Engr. Adolfo Portocarrero Reséndiz

Email: adolfo.portocarrero@conagua.gob.mx

Address: Av. Observatorio 192, Col. Observatorio, Del. Miguel Hidalgo. C.P. 11860, México D.F. Tel. (55) 2636-4600 Servicio Meteorológico Nacional

1. UNAM network

| Document Name:  Vantage Pro2. Manual de la consola. Para las estaciones meteorológicas Vantage Pro2™ y Vantage Pro2 Plus™ |
| --- |
| Data Source: <https://www.ruoa.unam.mx/pembu/index.php?page=data_sheet> |
| Last Access Date: 05/27/2020 |
| Document Name:  Conjunto de sensores integrado. Para las estaciones meteorológicas Vantage Pro2™ y Vantage Pro2™ Plus. |
| Data source: <https://www.ruoa.unam.mx/pembu/index.php?page=data_sheet> |
| Last Access Date: 05/27/2020 |

- 1. General criteria for selecting the location of the integrated sensor suite

The location of the monitoring stations in each UNAM high school depends on their particular conditions. Access to electricity and the Internet is continuously available for proper operation. Most of the stations are located on the school roofs, most of which have terracotta-colored waterproofing coating. Maintenance is carried out at least twice a year: the first period from February to April and the second period from September to November of each year (Personal communication). Meteorological stations located in the UNAM monitoring network use the wireless Vantage Pro2 Plus type instrument, DAVIS brand, with WeatherLink software and USB connection.

- 1. Location and exposure of instruments

Temperature

- The temperature sensors are mounted on a passive solar radiation shield to reduce the impact of solar radiation on the sensor readings and follow the next specifications:
- Located away from the influence of heat sources such as chimneys and gas vents and at least 30m away from any concrete or asphalt road that absorbs and radiates solar heat during the day.
- Avoid locations near or on top of objects or areas that absorb and radiate solar heat during the day like pavement, terraces, metal or concrete structures.
- Installation near water features such as swimming pools or ponds is avoided.

Wind speed

- Anemometer is placed at least 2 m above any surrounding obstructions (trees or buildings) and at the top of 9m height towers.
  1. Contact information

MSc. Enrique Azpra Romero

Red Universitaria de Observatorios Atmosféricos (RUOA)

email: ear1@atmosfera.unam.mx

Phone: (55) 5623 8222 ext. 44974

1. REDMET network

| Document Name:  La Red Meteorológica (REDMET) y telemetría del Sistema de Monitoreo Atmosférico de la ciudad de México (SIMAT) |
| --- |
| Data Source: <http://www.ptolomeo.unam.mx:8080/jspui/bitstream/132.248.52.100/7963/1/Informe%20de%20actividades%203.pdf> |
| Last Access Date: 05/27/2020 |

The quality control system of the REDMET network operates under the regulations of national and international entities such as the WMO and EPA to carry out the calibration methods of its instruments.

Each monitoring station that integrates the REDMET owns a data acquirer and electronic communication system for the transmission of the information generated on-site to the REDMET computer center.

- 1. Technical specifications of sensors for temperature and wind speed

Temperature sensor

- REDMET has two different models of sensors for ambient temperature including the Met One brand sensors model 083E-1-6 and model HMP60.
- These sensors are installed inside a solar radiation shield, mounted on the meteorological tower at 4 m above the base of the station. Sensor has an armor (plate type) with forced or aspirated air.

Wind speed sensor

- REDMET has two types of electromechanical wind speed sensors, the dual sensor (wind speed and direction) model Met One 034B, and the individual sensors, Met One 010C and Met One- 020C.
- Wind speed is represented as a selectable scalar speed in units (m/s, mph, km/h).
  1. Contact information

Sistema de Monitoreo Atmosférico de la Ciudad de México (SIMAT)

email: [calidadaire@sedema.cdmx.gob.mx](mailto:calidadaire@sedema.cdmx.gob.mx)

Phone: 5278-9931 ext. 6260

Address: Av. Tlaxcoaque # 8, Piso 6, Col. Centro, C.P. 06090, Del. Cuauhtémoc, CDMX.

1. Weather Underground network

| Station Name | Surface Type | Station type | Software |
| --- | --- | --- | --- |
| IALMOLOY3 | composite-shingles | Davis Vantage Pro2 (Wireless) | weatherlink.com 1.10 |
| IARAMB3 | cement | AcuRite 5-in-1 Weather Station with Wi-Fi | NA |
| ICIUDADD99 | shrubbery | AcuRite Pro Weather Center | myAcuRite |
| ICIUDADL14 | gravel | Ambient Weather WS-2902 | AMBWeatherV4.0.2 |
| ICOACALC3 | composite-shingles | Ambient Weather WS-1400-IP (Wireless) | Weather logger V3.0.5 |
| ICORONAN2 | cement | Ambient Weather WS-2902 | AMBWeatherV4.0.2 |
| IFRACCIO2 | grass | AcuRite Pro Weather Center | myAcuRite |
| IIXCAQUI2 | composite-shingles | Davis Vantage Pro2 (Wireless) | weewx-3.5.0 |
| ILERMA2 | composite-shingles | Davis Vantage Pro2 Plus (Cabled) | weewx-3.8.0 |
| IMEXICOC50 | rooftop (wood shingles) | AcuRite 5-in-1 Weather Station with AcuRite Access | myAcuRite |
| IMEXICOC47 | composite-shingles | Ambient Weather WS-1001-WiFi (Wireless) | WS-1001 V2.2.9 |
| IMEXIC1 | rooftop (composite-shingles) | AcuRite Pro Weather Center | myAcuRite |
| IMEXICOC46 | composite-shingles | Ambient Weather WS-2090 (Wireless) | EasyWeather V8.8.0 |
| IMEXICOC34 | spanish-tiles | Davis Vantage Vue (Wireless) | weatherlink.com 1.10 |
| IMEXICOC35 | cement | Netatmo Weather Station | http://meteoware.com |
| IMEXICOC44 | spanish-tiles | Ambient Weather WS-2000 | AMBWeatherV4.0.2 |
| IMEXICOC40 | spanish-tiles | Ambient Weather WS-2902 | AMBWeatherV3.0.3 |
| IMEXICOC29 | composite-shingles | Netatmo Weather Station | WeatherApp |
| IMEXICOC48 | other | AcuRite Atlas Weather Station with AcuRite Access | myAcuRite |
| IMIGUELH4 | composite-shingles | Davis Vantage Pro2 (Wireless) | meteobridge |
| INAUCALP34 | composite-shingles | Ambient Weather WS-2902 | AMBWeatherV4.0.3 |
| IPUEBLAC5 | spanish-tiles | Ambient Weather WS-900-IP (Wireless) | Weather logger V3.1.2 |
| IPUEBLAC11 | composite-shingles | Davis Vantage Pro2 Plus (Cabled) | weatherlink.com 1.10 |
| IPUEBLAC13 | composite-shingles | Davis Vantage Pro2 Plus (Cabled) | weatherlink.com 1.10 |
| IPUEBLAC14 | composite-shingles | Davis Vantage Pro2 Plus (Cabled) | weatherlink.com 1.10 |
| IPUEBLAC15 | composite-shingles | Davis Vantage Pro2 Plus (Cabled) | weatherlink.com 1.10 |
| IPUEBL5 | other | other | WH2600GEN_V2.2.5 |
| ITECOZAU2 | composite-shingles | AcuRite Pro Weather Center | myAcuRite |
| ITEPEACA2 | grass | Ambient Weather WS-2090 (Wireless) | AMBWeatherV3.0.3 |
| IVALLEDE36 | composite-shingles | Ambient Weather WS-2902 | NA |
| IDFMEXIC11 | composite-shingles | Davis | weatherlink.com 1.10 |
| IDISTRIT45 | composite-shingles | Davis Vantage Pro2 (Cabled) | Weather logger V3.1.0 |
| IDISTRIT69 | cement | Netatmo Weather Station | WeatherApp |
| IESTADOD44 | grass | Ambient Weather WS-1400-IP (Wireless) | WH2602 V4.5.8 |
| IESTADOD4 | trees | Davis Vantage Pro2 (Cabled) | weatherlink.com 1.10 |
| IESTADOD6 | spanish-tiles | Netatmo Weather Station | http://meteoware.com |
| IESTADOD2 | grass | Davis Vantage Pro2 Plus | Wunderground v.1.15 PWSDec 27 2007 |
| IGUERRER8 | spanish-tiles | Davis Vantage Vue (Wireless) | weatherlink.com 1.10 |
| IJALISCO24 | NA | Davis Vantage Pro 2 | weatherlink.com 1.10 |
| IMORELOS8 | composite-shingles | AcuRite Pro Weather Center | myAcuRite |
| IMORELOS9 | composite-shingles | Davis Vantage Vue (Wireless) | weatherlink.com 1.10 |
| IJALISCO19 | cement | Davis Vantage Pro2 Plus (Wireless) | weatherlink.com 1.10 |
| IMORELOS7 | cement | Ambient Weather WS-1001-WiFi (Wireless) | WS-1001 V2.2.2 |
| IPUEBLAP18 | trees | Ambient Weather WS-1001-WiFi (Wireless) | WS-1001 V2.1.9 |
| IPUEBLAP12 | composite-shingles | Davis Vantage Pro2 (Cabled) | weatherlink.com 1.10 |
| IPUEBLAP8 | composite-shingles | Davis Vantage Pro Plus | weatherlink.com 1.10 |
| IPUEBLAP9 | composite-shingles | Davis Vantage Pro2 (Cabled) | weatherlink.com 1.10 |
| IPUEBLAP4 | NA | Davis Vantage Pro | weewx-3.9.1 |
| IPUEPUEB2 | NA | Davis Vantage Pro | Wunderground v.1.15 PWSDec 27 2007 |
| IPUEBLAT6 | composite-shingles | Vantage Pro2 | weatherlink.com 1.10 |
| IHUIMILP1 | NA | VANTAGE PRO 2 PLUS | Wunderground v.1.15 PWSDec 27 2007 |
| IQUERETA19 | composite-shingles | VANTAGE PRO 2 PLUS | Wunderground v.1.15 PWSDec 27 2007 |
| IQUERETA10 | NA | VANTAGE PRO 2 PLUS | Wunderground v.1.15 PWSDec 27 2007 |
| IQUERETA17 | composite-shingles | VANTAGE PRO 2 PLUS | weatherlink.com 1.10 |
| IQUERETA23 | NA | VANTAGE PRO PLUS | Wunderground v.1.15 PWSDec 27 2007 |
| IQUERETA13 | NA | VANTAGE PRO 2 PLUS | Wunderground v.1.15 PWSDec 27 2007 |
| IQUERETA29 | composite-shingles | Davis Vantage Pro2 Plus (Cabled) | weatherlink.com 1.10 |
| ISTATEOF3 | grass | Davis Vantage Pro2 Plus (Wireless) | Wunderground v.1.15 PWSDec 27 2007 |
| ITLAXCAL3 | NA | Davis Pro | weatherlink.com 1.10 |
| ICIUDA6 | cement | AcuRite 5-in-1 Weather Station with AcuRite Access | NA |
| IGENER13 | cement | AcuRite 5-in-1 Weather Station with AcuRite Access | myAcuRite |
| IJOJUT4 | rooftop (spanish tiles) | Ambient Weather WS-2902 | AMBWeatherV4.2.8 |
| IMEXICOC30 | cement | Davis Vantage Pro2 Plus (Wireless) | weatherlink.com 1.10 |
| INAUCALP28 | cement | Ambient Weather WS-900-IP (Wireless) | EasyWeather V8.8.0 |
| IPUEBLAC19 | composite-shingles | AcuRite 5-in-1 Weather Station with Wi-Fi | NA |
| ISANCR8 | rooftop (composite-shingles) | AcuRite 5-in-1 Weather Station with AcuRite Access | myAcuRite |
| ITEHUI2 | NA | Davis Vantage Pro2 (Wireless) | weewx-3.5.0 |
| IPUEBLAP11 | composite-shingles | Davis Vantage Pro2 (Cabled) | weatherlink.com 1.10 |
| IAMEALCO2 | NA | VANTAGE PRO 2 PLUS | Wunderground v.1.15 PWSDec 27 2007 |
| IQUERETA11 | NA | Vantage Pro 2 Plus | Wunderground v.1.15 PWSDec 27 2007 |
